# PD-L1-expressing cancer-associated fibroblasts induce tumor immunosuppression and contribute to poor clinical outcome in esophageal cancer

**DOI:** 10.1007/s00262-023-03531-2

**Published:** 2023-09-05

**Authors:** Kento Kawasaki, Kazuhiro Noma, Takuya Kato, Toshiaki Ohara, Shunsuke Tanabe, Yasushige Takeda, Hijiri Matsumoto, Seitaro Nishimura, Tomoyoshi Kunitomo, Masaaki Akai, Teruki Kobayashi, Noriyuki Nishiwaki, Hajime Kashima, Naoaki Maeda, Satoru Kikuchi, Hiroshi Tazawa, Yasuhiro Shirakawa, Toshiyoshi Fujiwara

**Affiliations:** 1 Department of Gastroenterological Surgery, Okayama University Graduate School of Medicine, Dentistry and Pharmaceutical Sciences, 2-5-1 Shikata-cho, Kita-ku, Okayama, 700-8558 Japan; 2 Department of Pathology and Experimental Medicine, Okayama University Graduate School of Medicine, Dentistry and Pharmaceutical Sciences, Okayama, Japan; 3 Center for Innovative Clinical Medicine, Okayama University Hospital, Okayama, Japan; 4 Department of Surgery, Hiroshima City Hiroshima Citizens Hospital, Hiroshima, Japan

**Keywords:** Esophageal cancer, Cancer-associated fibroblasts, Programmed cell death 1, Program cell death ligand 1, Immune checkpoint inhibitors

## Abstract

The programmed cell death 1 protein (PD-1)/programmed cell death ligand 1 (PD-L1) axis plays a crucial role in tumor immunosuppression, while the cancer-associated fibroblasts (CAFs) have various tumor-promoting functions. To determine the advantage of immunotherapy, the relationship between the cancer cells and the CAFs was evaluated in terms of the PD-1/PD-L1 axis. Overall, 140 cases of esophageal cancer underwent an immunohistochemical analysis of the PD-L1 expression and its association with the expression of the α smooth muscle actin, fibroblast activation protein, CD8, and forkhead box P3 (FoxP3) positive cells. The relationship between the cancer cells and the CAFs was evaluated in vitro, and the effect of the anti-PD-L1 antibody was evaluated using a syngeneic mouse model. A survival analysis showed that the PD-L1^+^ CAF group had worse survival than the PD-L1^-^ group. In vitro and in vivo, direct interaction between the cancer cells and the CAFs showed a mutually upregulated PD-L1 expression. In vivo, the anti-PD-L1 antibody increased the number of dead CAFs and cancer cells, resulting in increased CD8^+^ T cells and decreased FoxP3^+^ regulatory T cells. We demonstrated that the PD-L1-expressing CAFs lead to poor outcomes in patients with esophageal cancer. The cancer cells and the CAFs mutually enhanced the PD-L1 expression and induced tumor immunosuppression. Therefore, the PD-L1-expressing CAFs may be good target**s** for cancer therapy, inhibiting tumor progression and improving host tumor immunity.

**Supplementary Information:**

The online version contains supplementary material available at 10.1007/s00262-023-03531-2.

## Introduction

Esophageal cancer is one of the most dangerous malignant tumors [1]. The 5-year survival rates of patients treated with endoscopic resection, surgery, concurrent chemoradiotherapy, or radiotherapy alone are 86.0%, 54.5%, 28.1%, and 26.5%, respectively [2]. Recently, esophageal cancer has been treated with multidisciplinary therapy consisting of surgery, chemotherapy, radiotherapy, and immunotherapy [3]. Immunotherapy has been successfully applied in clinical practice as a novel therapeutic approach; however, there are problems, including low response rates, acquired resistance, and immune-related adverse events [4]. Furthermore, owing to the heterogeneity within the immune microenvironment and various oncological characteristics, the exact mechanism of immunotherapeutic refractory remains unclear [4]. Therefore, evaluating the tumor microenvironment (TME) is vital for achieving better therapeutic efficacy [5].

The TME comprises various cell types, including cancer cells, inflammatory cells, blood vessels, extracellular matrix, and cancer-associated fibroblasts (CAFs). CAFs are abundant and vital components of TME [6]. Since CAFs are a heterogeneous population and play a key role in tumor-promoting functions via paracrine signaling and direct physical interactions, further functional analysis and potential as therapeutic targets have been explored [7, 8]. Previously, we reported the tumor-promoting functions of CAFs in angiogenesis, therapeutic resistance, invasion, migration, lymph node metastasis, and tumor immunosuppression [9–12]. Furthermore, we demonstrated that α smooth muscle actin (αSMA) and fibroblast activation protein (FAP), which are used as CAFs markers, are poor survival factors for clinical specimens of esophageal cancer [11, 12]. Regarding the immunosuppressive functions, it has also been reported that cytotoxic T cells are attenuated; in contrast, regulatory T cells (Tregs) are promoted via interleukin 6 (IL6) secreted from CAFs [12].

Programmed cell death 1 (PD-1) on the T-cell surface binds to programmed cell death ligand 1 (PD-L1), resulting in the inhibition of immune responses and promotion of self-tolerance [13]. Several cancer cells express PD-L1 and escape the antitumor response and tumor-promoting system via the PD-1/PD-L1 axis [14, 15]. High PD-L1 expression has been reported as a poor prognostic factor for various solid tumors [13, 16, 17]. Recent clinical trials have revealed that immune checkpoint inhibitors (ICIs) contribute to better survival rates than conventional chemotherapy, which led to the approval of ICIs for treating esophageal cancer by the United States Food and Drug Administration. Therefore, the clinical indications for ICIs, including the targeting of the PD-1/PD-L1 axis, are dramatically expanding. However, a minority of patients achieve sustained durable remission [18, 19]. The response rate to ICIs for esophageal cancer is 9.9%–30%, which is not necessarily high [20].

In addition, CAFs induce the expression of the immune checkpoint molecule PD-1 on T cells and PD-L1 on cancer cells [21, 22]. However, it is unclear how cancer cells and CAFs are involved in the PD-1/PD-L1 axis within tumors. High expression levels of PD-L1 in cancer cells and tumor-infiltrated immune cells, defined as a combined proportion score (CPS), induce more efficacy of ICIs therapy, suggesting its role as a molecular biomarker [23]. Recently, a population of PD-L1-expressing CAFs was reported [21, 24]. However, the clinical significance of PD-L1-expressing CAFs remains controversial, owing to the limited evidence in various tumors. In addition, the role of PD-L1-expressing CAFs in ICIs therapy remains unclear. Therefore, the impact of PD-L1-expressing CAFs on TME and ICIs therapy should be examined to overcome the low response rate in clinical practice.

To investigate the relationship between CAFs and the PD-1/PD-L1 axis, we hypothesized that PD-L1-expressing CAFs are present in esophageal cancer and that they have an immunosuppressive function, resulting in aggressive tumors. Furthermore, we explored potential therapeutic targets for PD-L1-expressing CAFs. Therefore, we report the impact of PD-L1-expressing CAFs using clinical specimens of patients with esophageal cancer and the efficacy of PD-L1 blockade for tumors with PD-L1-expressing CAFs in syngeneic murine models.

## Materials and methods

### Patients and clinical information

We retrospectively reviewed 140 patients who underwent radical esophagectomy with lymph node dissection at the Department of Gastroenterological Surgery of Okayama University Hospital from 2008 to 2010. The exclusion criteria were as follows: (i) esophagectomy after endoscopic mucosal resection or endoscopic submucosal dissection; (ii) pathological diagnosis of melanoma; (iii) distant metastasis; (iv) complete response after neoadjuvant chemotherapy; and (v) unevaluable tumor. The tumor classification was applied to the tumor-node-metastasis (TNM) Classification of Malignant Tumors, 7th edition, established by the Union for International Cancer Control (UICC).

### Immunohistochemistry of clinical specimens

The staining details for αSMA, CD8, and FoxP3 have been previously reported [12]. The slides were stained with CD8 (clone C8/144B, Dako, Glostrup, Denmark 1:100 dilution), FoxP3 (ab20034, clone 236A/E7, Abcam, Cambridge, UK, 1:100 dilution), and αSMA (A2547, clone 1A4, Sigma-Aldrich, St. Louis, MO, USA, 1:1000 dilution). Briefly, the presence of tumor tissue was firstly confirmed by hematoxylin and eosin (HE) staining. Next, for the immunohistochemistry, sections were incubated with primary antibody against FAP (ab207178, clone EPR20021, Abcam, 1: 250 dilution) for 60 min at room temperature (RT) and against PD-L1 (#13684, clone E1L3N, Cell Signaling Technology, Danvers, MA, USA, 1: 200 dilution) overnight at 4℃. After incubation with the primary antibody, the sections were incubated with a secondary antibody (K4003, Dako EnVision + System-HRP Labelled Polymer Anti-Rabbit, Dako) for 30 min at RT. A Dako Liquid DAB^+^ Substrate Chromogen System (K3468, Dako) was applied to each section for visualization. They were photographed using a microscope (BX51; Olympus, Tokyo, Japan).

### Immunohistochemical analysis of clinical samples

The numbers of cells expressing CD8 or FoxP3 and the αSMA score were measured as reported previously [12]. The FAP score was calculated as an area index using the ImageJ software (http://rsb.info.nih.gov/ij/). The evaluation method for PD-L1 was described as follows. First, three representative areas were selected under high magnification. The number of PD-L1-expressing cancer cells and total cancer cells was counted in the field. PD-L1 expression in cancer cells was defined by partial or complete cell membrane staining. Cancer cells where only the cytoplasm was stained were considered to be negative. The proportion score of PD-L1 was defined as the percentage of PD-L1-expressing cancer cells over the total number of tumor cells in the denominator. A cutoff value of 10% was set for the PD-L1^+^ cancer cell group. Also, we defined PD-L1^+^CAFs as neither cancer cells nor immune cells, but spindle-shaped, cells in the stroma with stained cytoplasm or cell membrane in PD-L1 immunohistochemistry. If spindle-shaped cells in the stroma area were expressed with PD-L1, the cases were considered as the PD-L1^+^ CAFs group. PD-L1^−^ cancer cells and PD-L1^−^ CAFs group were indicated as double negative; PD-L1^+^ cancer cells and PD-L1^−^ CAFs group were indicated as cancer single positive; PD-L1^−^ cancer cells and PD-L1^+^ CAFs group were classified as CAFs single positive; PD-L1^+^ cancer cells and PD-L1^+^ CAFs group were indicated as double positive.

### Immunofluorescence microscopy

Deparaffinized tissue sections were incubated with primary antibodies against human PD-L1 (#13684, clone E1L3N, Cell Signaling Technology, 1: 200 dilution) or digoxigenin (#700772, clone 9H27L19, Thermo Fisher Scientific, Waltham, MA, USA, 1: 500 dilution) overnight at 4℃. Next, the sections were incubated with the secondary antibody (#A21069, Alexa Fluor® 568 F(ab’)_2_ fragment of goat anti-rabbit IgG (H + L), Thermo Fisher Scientific) for 30 min at RT. After washing, the sections were incubated with FITC-labeled anti-αSMA antibody (ab8211, clone 1A4, Abcam, 1: 100 dilution) overnight at 4℃. The sections were mounted with coverslips and mounting medium containing DAPI (P36981; ProLong Glass Antifade Mountant, Thermo Fisher Scientific); subsequently, they were photographed using a fluorescence microscope (IX83; Olympus).

### Cell lines

Human esophageal squamous cell carcinoma (TE4 and TE8) and esophageal adenocarcinoma (OE33) cell lines were used. TE4 and OE33 cells were purchased from the Japanese Collection of Research Bioresources Cell Bank (Osaka, Japan), while TE8 was purchased from the RIKEN BRC Cell Bank (Tsukuba, Japan). Murine colon adenocarcinoma (MC38) was purchased from Kerafast (Boston, MA, USA), and Yuta Shibamoto (Department of Quantum Radiology, Nagoya City University, Nagoya, Japan) kindly provided murine dermal squamous cell carcinoma (SCCVII) cell line. Primary human esophageal fibroblasts, designated as FEF3, were isolated from the human fetal esophagus, as previously described [9]. Murine fibroblasts (MEF) were purchased from the American Type Culture Collection (Manassas, VA, USA). TE4, TE8, and OE33 cells were maintained in RPMI-1640 medium (FUJIFILM, Tokyo, Japan) supplemented with 10% fetal bovine serum (FBS), 100 units/mL penicillin, and 100 µg/mL streptomycin. SCCVII and FEF3 cells were maintained in Dulbecco’s modified Eagle’s medium (DMEM, FUJIFILM) supplemented with 10% FBS, 100 units/mL penicillin, and 100 µg/mL streptomycin. MEFs were maintained in DMEM supplemented with 15% FBS, 100 units/mL penicillin, and 100 µg/mL streptomycin. MC38 cells were maintained in DMEM supplemented with 10% FBS, 2 mM glutamine, 0.1 mM nonessential amino acids, 1 mM sodium pyruvate, 10 mM Hepes, 50 µg/mL gentamicin sulfate, 100 units/mL penicillin, and 100 µg/mL streptomycin. All cells were maintained at 37℃ in a 5% CO_2_ incubator. After thawing, the cells were cultured for no more than 20 passages.

### Activation of cancer cells and fibroblasts

Fibroblasts were cultured in DMEM supplemented with 10% FBS for 48 h, and cancer cells were cultured in DMEM supplemented with 2% FBS for 48 h to produce conditioned medium (CM) by fibroblasts or cancer cells. Subsequently, the culture supernatants were collected, centrifuged at 1000 rpm for 5 min, and preserved at -30℃ as conditioned media of fibroblasts and cancer cells, respectively. These cells were cultured in different CM for 72–96 h (e.g., cancer cells were cultured with CM made from fibroblasts) to activate cancer cells or fibroblasts. Also, human fibroblasts were incubated and stimulated for 72 h using human transforming growth factor β1 (TGF-β1, HZ-1011, Proteintech Group, Inc., Rosemont, IL, USA), and murine TGF-β1 (7666-MB-005, R&D Systems, Minneapolis, MN, USA). These cells were collected and used as stimulated cells. Fibroblasts activated using TGF-β were indicated as MEF TGF-β, FEF3 TGF-β, and CM of cancer cells; FEF3 CM-TE4, FEF3 CM-TE8, and FEF3 CM-OE33.

### Flow cytometry analysis

Single-cell suspension was obtained as previously described [25]. The cells were stained with following antibodies; APC-labeled anti-human PD-L1 antibody (#329707, clone 29E.2A3, BioLegend, San Diego, CA, USA), APC-labeled anti-mouse PD-L1 antibody (#124311, clone 10F.9G2, BioLegend), FITC-labeled anti-CD45 (#103107, clone 30-F11, BioLegend), monoclonal PerCP/Cyanine5.5-labeled anti-CD31 (#102419, clone 390, BioLegend), monoclonal PE-labeled anti-CD90.2 (#105307, clone 30-H12, BioLegend), human IgG isotype control antibody (#400322, clone MPC-11, BioLegend), and murine-IgG isotype control antibody (#400612, clone RTK4530, BioLegend). Red blood cell lysis buffer (420302, BioLegend) and Debris Removal Solution (130-109-398, Miltenyi Biotec, Bergisch Gladbach, Germany) were also used. Dead cells (1:1000 dilution) were stained using a Zombie NIR Fixable Viability Kit (423106, BioLegend). Stained cells were analyzed using flow cytometry (FACSLyric; BD Biosciences, Franklin Lakes, NJ, USA), and data were analyzed using the FlowJo software (BD Biosciences).

### Co-culture model

Cytotell UltraGreen dye (22240, AAT Bioquest, Sunnyvale, CA, USA) was used as a pre-labeled fibroblast. Fibroblasts (0.5 × 10^6^) were resuspended in 500 µL of the CytoTell UltraGreen dye working solution and incubated for 30 min at 37 ℃ in darkness. Cancer cells (0.1 × 10^6^) and pre-labeled fibroblasts (0.1 × 10^6^) were co-cultured directly in six-well plates for 72 h. Co-cultured cancer cells and pre-labeled fibroblasts were analyzed for PD-L1 expression using flow cytometry.

### Animal study

Five-week-old female C57BL/6 and C3H/He mice were purchased from Clea (Tokyo, Japan). MC38 (0.5 × 10^6^) cells alone or MC38 (0.5 × 10^6^) cells with MEF (0.5 × 10^6^) were inoculated into the subcutaneous right flank of C57BL/6 mice. SCCVII (0.5 × 10^6^) cells alone or SCCVII (0.5 × 10^6^) cells with MEF (0.5 × 10^6^) cells were inoculated into the subcutaneous right flank in C3H/He mice. MC38 or SCCVII alone (cancer cell-alone group) and MC38 or SCCVII inoculated with MEF (co-inoculated group) were defined. Tumor volume (mm^3^) was calculated every 3 days using the following formula: length × width^2^ × 0.5. Mice were randomly categorized into two groups to avoid differences when the tumors reached 50 mm^3^. Treatment with 50 μg/body of anti-PD-L1 antibody (BE0101, clone 10F.9G2, BioXCell, Lebanon, NH, USA) and 50 μg/body of isotype control rat IgG2b (BE0090, clone LTF-2, BioXCell) was administered intraperitoneally every 3 days. In the anti-PD-L1 antibody administration experiment, the tumors were harvested 3 days after the last dose. The mice were euthanized by inhalation of CO_2_ when the tumor volume reached 1000 mm^3^.

### Immunohistochemistry in allograft models

The protocol of harvested tumors was previously described [12]. The following antibodies were used; CD8a (#14-0808-82, clone 4SM15, eBioscience, San Diego, CA, USA, 1: 100 dilution, for 60 min at RT), FoxP3 (#14-5773-82, clone FJK-16s, eBioscience, 1: 100 dilution, for 60 min at RT), αSMA (A5228, clone 1A4, Sigma-Aldrich, 1:1000 dilution), and digoxigenin (#700772, clone 9H27L19, Thermo Fisher Scientific, Waltham, MA, USA, 1: 500 dilution, overnight at 4 ℃). Each section was counterstained using Mayer’s hematoxylin. The number of CD8^+^ or FoxP3^+^ cells and the area index of αSMA were calculated using the ImageJ software.

### Synthesis of digoxigenin-conjugated PD-L1 antibody

Digoxigenin (A2952, Thermo Fisher Scientific) was conjugated to a monoclonal anti-PD-L1 antibody (BE0101, clone 10F.9G2, BioXCell) and rat IgG2b (BE0090, clone LTF-2, BioXCell). For the protein labeling reaction, anti-PD-L1 antibody (1 mg) or rat IgG2b (1 mg) was mixed with digoxigenin (19.5 μg) suspended in dimethylsulfoxide in 0.3 mol/L Na_2_HPO_4_ (pH 8.5) for 2 h at RT. The mixture was purified on a PD-10 column (17085101; Cytiva, Tokyo, Japan).

### Statistical analysis

Overall survival (OS) and relapse-free survival (RFS) were analyzed using the Kaplan–Meier with the log-rank test. Hazard ratios were calculated using Cox proportional hazards regression in univariate and multivariate analyses. For the analysis of clinical specimens, proportions of categorical and continuous variables were compared using Fisher’s exact and Mann–Whitney *U* tests, respectively. Logistic regression analysis was performed to identify risk factors for the PD-L1^+^ group. Student’s *t*-test or ratio paired *t*-test was used for two-group comparisons of in vitro and in vivo experiments. Statistical significance was set at *P* < 0.05. All statistical analyses were performed using the EZR software (Saitama Medical Center, Jichi Medical University, Saitama, Japan) [26].

## Results

### Esophageal cancer patients with high PD-L1 expression in cancer cells had a poor survival

**Fig. 1:**
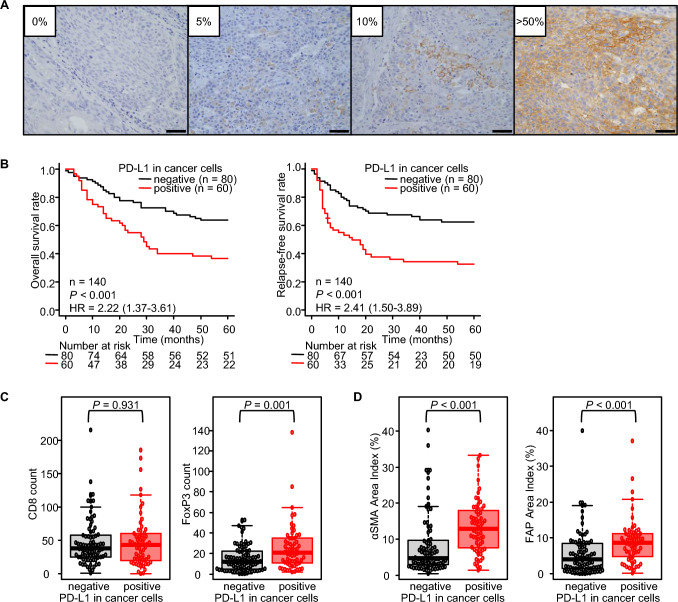
Immunohistochemistry focused on PD-L1 expression in cancer cells in clinical specimens for esophageal cancer. **A** Representative figures of each PD-L1 expression in cancer cells for esophageal cancer patients. Scale bars: 50 µm. **B** Survival analyses. **C** Comparison of immune cells between PD-L1^±^ cancer cells groups. **D** Comparison of CAFs between PD-L1^±^ cancer cell groups. (n = 140, **B**: Cox regression hazard model; HR, hazard ratio with 95% confidence intervals; **C**, **D**: Mann–Whitney U test)

To explore the correlation between PD-L1 overexpression and the outcome of patients with esophageal cancer, PD-L1 expression in resected tumors was evaluated by immunohistochemistry. Representative images of PD-L1 expression (0, 5, 10, and > 50% in whole cells) in esophageal cancers are shown in Fig. 1A. In this study, PD-L1^+^ cases were defined as tumors where > 10% of all cancer cells expressed PD-L1. 140 patients with esophageal cancer were analyzed, and 60 (42.9%) had PD-L1^+^ cancer cells. Regarding clinicopathological features, significant differences were observed in the pathological T stage and area index of αSMA and FAP between the PD-L1^±^ cancer cell groups (Supplemental Table S1). Survival analysis showed that the PD-L1^+^ cancer cell group had significantly worse OS and RFS than the PD-L1^−^ group (Fig. 1B). Furthermore, higher PD-L1^+^ cancer cells were independent predictive factors for OS (HR = 1.72, 95% CI 1.03–2.87, *P* = 0.039) and RFS (HR = 2.02, 95% CI 1.22–3.34, *P* = 0.006; Supplemental Table S2 and S3). In evaluating tumor immunity within the tumor bed, a relationship between high PD-L1^+^ cancer cells and the number of FoxP3^+^ Tregs was significantly detected. However, no correlation was observed with the number of CD8^+^ T cells (Fig. 1C). Additionally, PD-L1^+^ cancer cells were positively correlated with the expression of both αSMA and FAP (Fig. 1D). Moreover, the area index of αSMA was an independent risk factor for PD-L1^+^ cancer cells (OR = 4.72, 95% CI 1.81–12.30, *P* = 0.001; Supplemental Table S4). Therefore, these results demonstrated that cancer cells overexpressing PD-L1 were associated with a higher number of Tregs and CAFs within the tumors, resulting in poor outcomes in patients with esophageal cancer.

### PD-L1-expressing CAFs impacted the outcome of patients with esophageal cancer

**Fig. 2:**
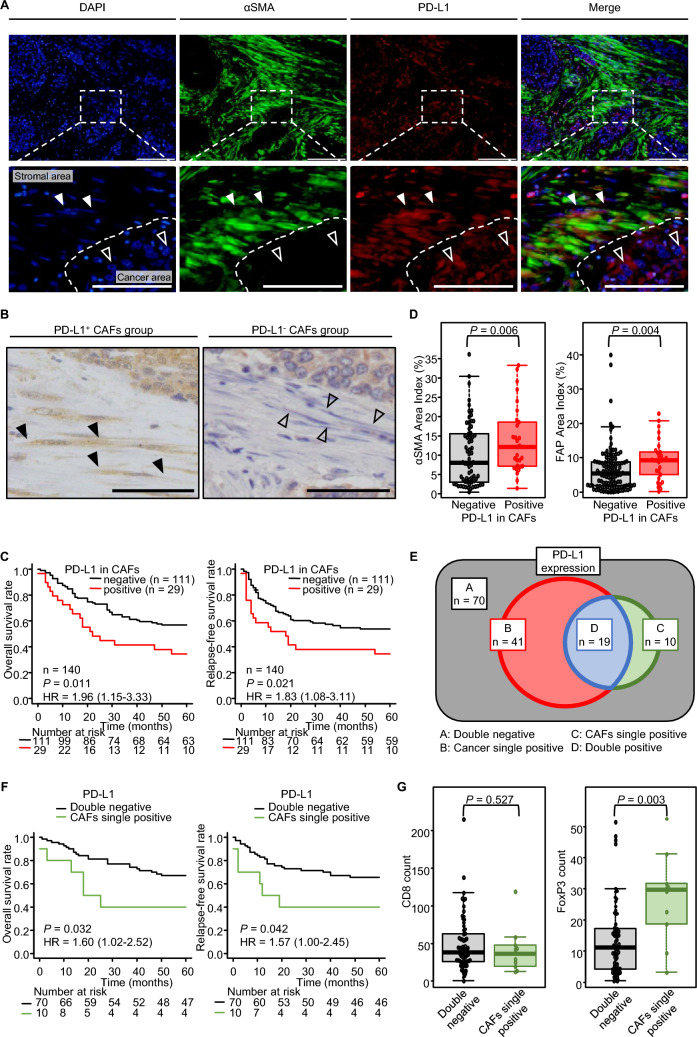
Immunohistochemistry focused on PD-L1 expression in CAFs in clinical specimens for esophageal cancer. **A** Representative figures of PD-L1 expression in the cancer area and stromal area. The filled arrowhead indicates CAFs, and the open arrowhead indicates cancer cells. Scale bars = 100 µm. Lower figures are enlarged images. Scare bars = 50 µm. **B** Representative picture of PD-L1^+^ CAFs (filled arrowhead) and PD-L1^−^ CAFs (open arrowhead). Scale bars = 50 µm. **C** Survival analyses (n = 140, Cox regression hazard model). **D** Comparison of CAFs between PD-L1^±^ groups (**C**, **D**; n = 140, Mann–Whitney *U* test). **E** The variance of PD-L1 expression was classified into four groups and organized using a Venn diagram. **F** Survival analysis for CAFs single positive versus double negative group (n = 80, Cox regression hazard model). **G** Comparison of immune cells between CAFs single positive and double negative group in PD-L1 expression (n = 80, Mann–Whitney *U* test). HR = hazard ratio with 95% confidence intervals

Regarding the types of PD-L1^+^ cells, immunofluorescence staining was conducted for the resected esophageal tumors. PD-L1 was expressed in both cancer and stromal cells (Fig. 2A). To evaluate resected specimens in esophageal cancer, spindle-shaped cells stained with PD-L1 in the stroma were defined as PD-L1-expressing CAFs (PD-L1^+^ CAF) using immunohistochemistry (Fig. 2B). PD-L1^+^ CAFs and PD-L1^−^ CAFs groups were defined as cases with or without the presence of PD-L1-expressing CAFs, respectively (Supplemental Figure S1). In the same clinical samples, immunohistochemical analysis showed that 29 (20.7%) patients had PD-L1^+^ CAFs. In OS and RFS, the PD-L1^+^ CAFs group had significantly worse outcomes than the PD-L1^−^ CAFs group (Fig. 2C). Next, we assessed the association of PD-L1-expressing CAFs with TME or tumor immunity factors. In host tumor immunity, patients with PD-L1-expressing CAFs had no relationship with CD8^+^ T cells (Supplemental Figure S2). In contrast, patients with PD-L1-expressing CAFs also showed significantly higher αSMA and FAP expression (Fig. 2D). The variance in PD-L1 expression was classified into four groups, and the outcome in esophageal cancer was analyzed (Fig. 2E and Supplemental Figure S3). Focusing on the groups without PD-L1-expressing cancer cells, the PD-L1^+^ CAFs group (CAFs single positive) had a significantly poorer OS and RFS than the PD-L1^−^ CAFs group (double negative; Fig. 2F). Furthermore, the CAFs single-positive group had significantly more Tregs than the double-negative group, whereas no correlation was found in CD8^+^ T cells between the two groups (Fig. 2G). The clinical specimens’ results showed that PD-L1-expressing CAFs were associated with poor outcomes.

### PD-L1 expression in fibroblasts was enhanced by stimulation of cancer cells

**Fig. 3:**
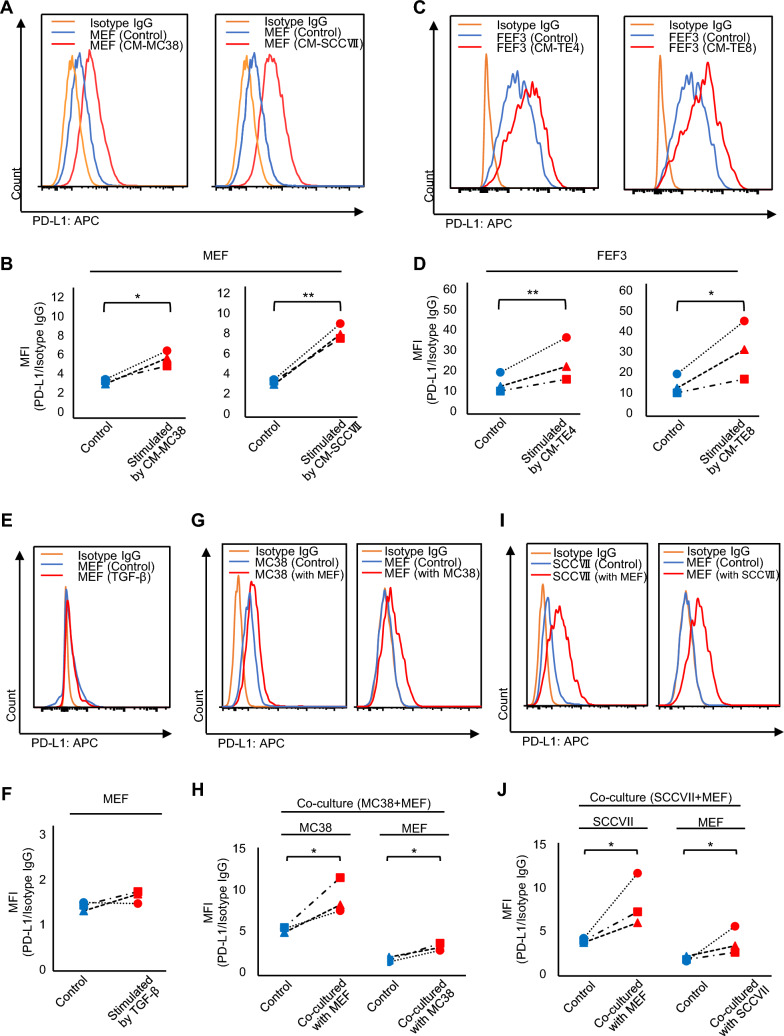
PD-L1 expression in fibroblasts and cancer cells in vitro. (A-D) Flow cytometry analysis of cell surface PD-L1 expression. **A** Histogram of PD-L1 expression and **B** comparison of PD-L1 expression in MEF with versus without stimulation by CM of MC38 or SCCVII. **C** Histogram of PD-L1 expression and **D** comparison of PD-L1 expression in FEF3 with versus without stimulation by CM of TE4 or TE8. **E**, **F** PD-L1 expression in MEF stimulated by TGF-β by flow cytometry. **E** Histogram of PD-L1 expression and **F** comparison of PD-L1 expression with versus without stimulation by TGF-β. **G**–**J** Flow cytometry analysis of cell surface PD-L1 expression in a co-culture model. **G** Histogram of PD-L1 expression and (H) comparison of MC38 and MEF co-culture model. **I** Histogram of PD-L1 expression and **J** comparison of SCCVII and MEF co-culture model. (n = 3, comparative analysis of MFIs by ratio paired* t*-test, ^*^P < 0.05; ^**^P < 0.01.)

To quantify the crosstalk between cancer cells and fibroblasts, the expression level of PD-L1 was verified using CM derived from murine cancer cells. Representative gating strategy of flow cytometry was shown (Supplemental Figure S4A). The increase in PD-L1 expression at the cell membrane level was significant using both CM-MC38 and CM-SCCVII (Fig. 3A, B). Next, to evaluate the interactions between cancer cells and fibroblasts, these cells were directly co-cultured in vitro*,* and fibroblasts were pre-labeled with fluorescence staining to distinguish them from cancer cells (Supplemental Figures S4B and S5). In the interaction of human-derived fibroblasts (FEF3) and human esophageal squamous cell carcinoma cells (TE4 or TE8), activation with both CM-TE4 and CM-TE8 also significantly increased PD-L1 expression in FEF3 (Fig. 3C, D). However, stimulation with CM from esophageal adenocarcinoma cells (OE33) barely increased PD-L1 expression (Supplemental Figure S6). Stimulation by TGFβ, one of the CAF-inducing factors, was not promote the PD-L1 expression in MEF and FEF3 cells (Fig. 3E, F, and Supplemental Figure S7). Co-culture with MEF cells and cancer cells (MC38 or SCCVII) significantly enhanced PD-L1 expression in both cells (Fig. 3G–J). In co-culture with FEF3 cells and TE4 or TE8 cells, PD-L1 expression in FEF3 cells was also significantly increased, however PD-L1 expression in cancer cells was not (Supplemental Figure S7). Additionally, co-culture with fibroblasts and OE33 cells barely increased PD-L1 expression in each cell (Supplemental Figure S6). These results suggest that both cancer cells and fibroblasts were complementarily activated, resulting in increased PD-L1 expression highly in mouse-derived cancer cell models compared to human-derived models.

### In vivo co-inoculation of cancer cells and CAFs enhanced PD-L1 expression

**Fig. 4:**
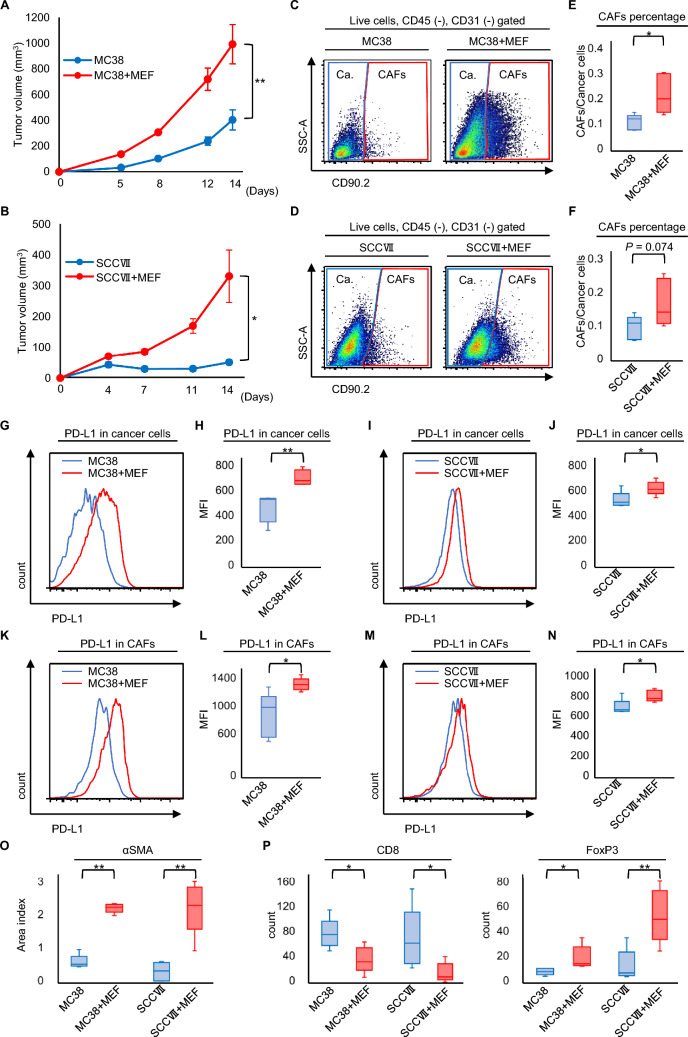
In vivo model of co-inoculation with cancer cells and fibroblasts, PD-L1 expression in both cancer cells and CAFs were evaluated. **A** Tumor growth of subcutaneous MC38 tumors with or without MEF (n = 5; Mean ± SEM. Student’s *t*-test). **B** Tumor growth of subcutaneous SCCVII tumors with or without MEF (n = 5; mean ± SEM. Student’s *t*-test). **C**, **D** Dot plot of flow cytometry identifying cancer cells (Ca.) (CD45^−^/CD31^−^/CD90.2^−^) and CAFs (CD45^−^/CD31^−^/CD90.2^+^) in the (C) MC38 and (D) SCCVII models. Dead cells were removal and subsequently gated out CD45 and CD31. The CD90.2 positive cells were identified as CAFs, while the CD90.2 negative cells were identified as cancer cells. **E**, **F** Evaluation of the CAF population is shown for each group [**E** MC38 or **F** SCCVII with or without MEF. n = 5, Student’s *t*-test]. **G**–**N** Histogram of PD-L1 expression in cancer cells for **G** MC38 and **I** SCCVII with versus without MEF tumor. Comparison of PD-L1 expression in cancer cells for **H** MC38 and **J** SCCVII with versus without MEF tumor. Histogram of PD-L1 expression in CAFs for **K** MC38 and **M** SCCVII with versus without MEF tumor. Comparison of PD-L1 expression in CAFs for **L** MC38 and **N** SCCVII with versus without MEF tumor (n = 5, comparative analysis of MFIs using Student’s *t*-test). **O** Comparison of the area index of αSMA at 400× magnification quantified using the ImageJ. (P) The average number of CD8-positive or FoxP3-positive T cells counted (n = 5, Student’s *t*-test). *P < 0.05; **P < 0.01

The impact of CAFs on cancer cells i*n vivo* was investigated using syngeneic mouse models. The tumor volume was significantly larger in the co-inoculation group than in the cancer cell-alone group in both MC38 and SCCVII models (Fig. 4A, B). The harvested tumors were analyzed using flow cytometry. (Fig. 4C, D). In both co-inoculation groups, the number of CAFs was higher than that in the cancer cell-alone group, implying that the co-inoculation tumor was a model of CAFs-rich tumors (Fig. 4E, F). Next, PD-L1 expression in cancer cells and CAFs was evaluated in the co-inoculation groups (Supplemental Figure S8). The mean fluorescence intensity (MFI) of PD-L1 in cancer cells was significantly increased in the co-inoculation groups in MC38 and SCCVII tumor models compared to the cancer cell-alone groups (Fig. 4G–J). Similarly, in both allograft models, PD-L1 expression in CAFs was also higher in the co-inoculated groups than in the cancer cell-alone groups (Fig. 4K–N). Furthermore, we evaluated the difference in immunogenicity between the two groups using immunohistochemistry (Supplemental Figure S9). Quantitative immunohistochemistry analyses also revealed increased αSMA expression in both the co-inoculated groups (Fig. 4O). Additionally, fewer CD8^+^ T cells and more Tregs were observed in the co-inoculation groups (Fig. 4P). These in vivo results showed that cancer cells and CAFs highly expressed PD-L1 in CAF-rich tumors, indicating an immune-suppressive tumor.

### Anti-PD-L1 antibody damaged cancer cells and CAFs in MC38 + MEF models, resulting in tumor immunity improvement

**Fig. 5:**
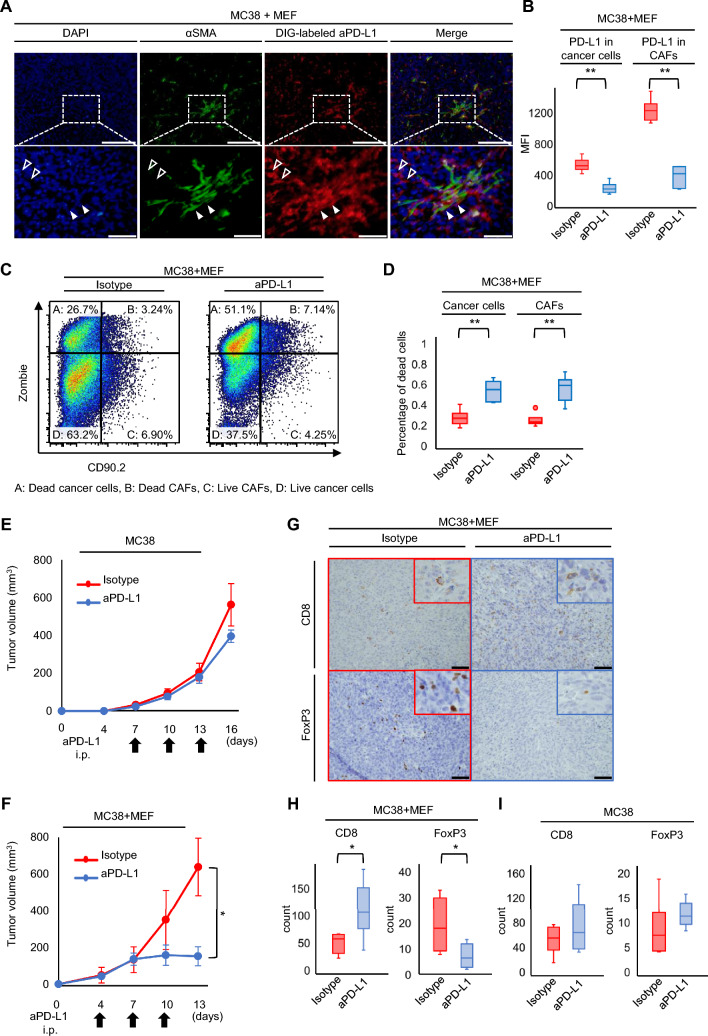
Administration of anti-PD-L1 antibody for co-inoculation model with MC38 cells and MEFs. **A** Multiple staining immunofluorescence images. The filled arrowhead indicates CAFs, and the open arrowhead indicates cancer cells. Scale bars = 200 µm. Lower figures are enlarged images. Scare bars = 50 µm. **B** Evaluations of PD-L1 expression in cancer cells and CAFs are shown in MC38 with MEF tumor after anti-PD-L1 antibody or isotype control (n = 6, comparative analysis of MFIs by Student’s *t*-test). **C** Representative figure of dot plot by flow-cytometric analysis for dead cells of cancer cells and CAFs. **D**, **E** Evaluations of dead cells in cancer cells and CAFs in MC38 with MEF tumor after aPD-L1 or Isotype control (n = 6, comparative analysis of the proportion of dead cells by Student’s *t*-test). **E**, **F** Tumor growth of subcutaneous MC38 tumors **F** with or **E** without MEF treated by anti-PD-L1 antibody or isotype control (n = 6; mean ± SEM. Student’s *t*-test). **G** Representative pictures of immunohistochemical staining for CD8 and FoxP3. Scale bars = 50 µm. **H**, **I** The average number of CD8^+^ or FoxP3^+^ T cells in MC38 tumors **H** with or **I** without MEF (n = 6, Student’s *t*-test). *P < 0.05, ^**^P < 0.01

First, the distribution of anti-PD-L1 antibodies in the co-inoculated groups was investigated to explore the effect of the anti-PD-L1 antibody utilizing the digoxigenin-labeled anti-PD-L1 antibody (DIG-PD-L1) in vivo. Immunofluorescence staining also showed that the DIG-PD-L1 stained αSMA^+^ cells, implying that anti-PD-L1 antibody could attach to PD-L1-expressing CAFs, similarly immunohistochemical staining (Fig. 5A and Supplemental Figure S10A). To evaluate the binding ability of the anti-PD-L1 antibody in the co-inoculated tumors, flow cytometric analysis was performed 24 h after administration. The MFI of PD-L1 was significantly reduced in both cancer cells and CAFs, suggesting successful binding of the anti-PD-L1 antibody to PD-L1-expressing cells (Fig. 5B). Moreover, 3 days after treatment with the anti-PD-L1 antibody, the percentage of dead cancer cells and CAFs was significantly increased compared with that in the control groups (Fig. 5C, D). These results indicate that treatment with the anti-PD-L1 antibody damaged both PD-L1-expressing cancer cells and CAFs. Next, the effects of anti-PD-L1 antibodies on tumor progression were evaluated. In the cancer cell-alone group, anti-PD-L1 antibody administration did not suppress tumor growth compared with isotype IgG (Fig. 5E). In contrast, in the MC38 + MEF group, the anti-PD-L1 group showed significantly suppressed tumor growth compared with the isotype group (Fig. 5F). Furthermore, tumor immunity was evaluated using tumor-infiltrating lymphocytes. In the MC38 + MEF model, CD8^+^ T cells were significantly increased, whereas Tregs were substantially decreased in the anti-PD-L1 antibody group (Fig. 5G, H). In the MC38 model, neither CD8^+^ T cells nor Tregs showed significant changes with the treatment (Fig. 5I and Supplemental Figure S11A). These results showed that the anti-PD-L1 antibody remarkably responded to CAFs-rich tumors and improved tumor immunity.

### Efficacy of anti-PD-L1 antibody for SCCVII + MEF tumor models

**Fig. 6:**
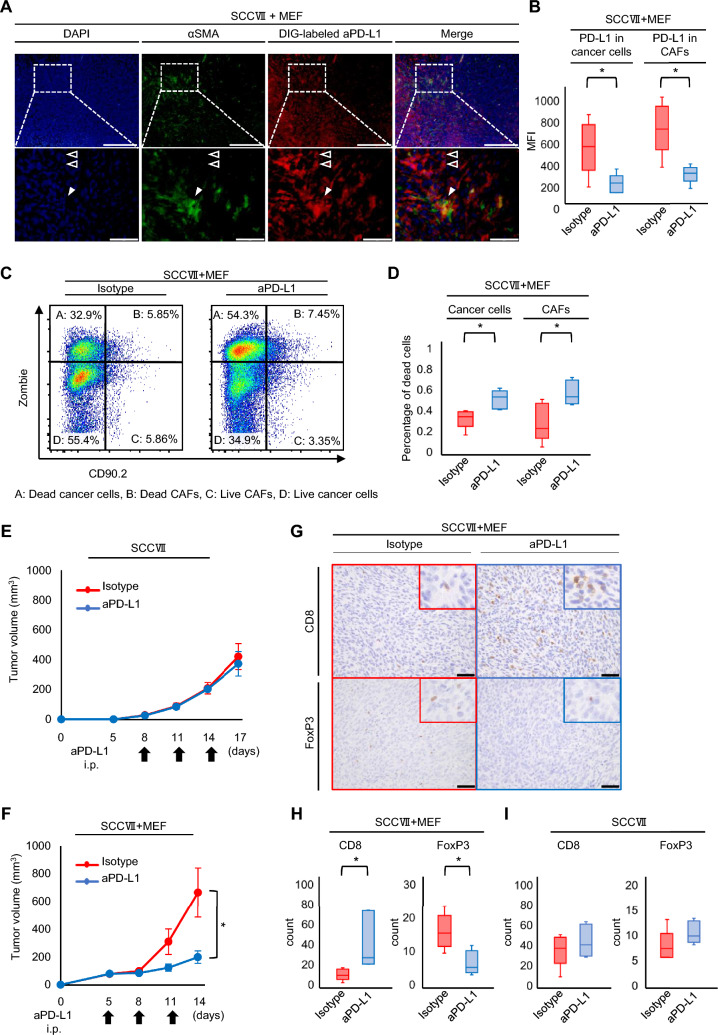
Administration of anti-PD-L1 antibody for co-inoculation model with SCCVII cells and MEFs. **A** Multiple staining immunofluorescence images of digoxigenin and αSMA. The filled arrowhead indicates CAFs, and the open arrowhead indicates cancer cells. Scale bars = 200 µm. Lower figures are enlarged images. Scare bars = 50 µm. **B** Evaluations of PD-L1 expression in cancer cells and CAFs are shown in MC38 with MEF tumor after anti-PD-L1 antibody or isotype (n = 6, comparative analysis of MFIs by Student’s *t*-test,). **C** Representative figure of dot plot by flow-cytometric analysis for dead cells of cancer cells and CAFs. **D** Evaluations of dead cells in cancer cells and CAFs in SCCVII with MEF tumor after aPD-L1 or Isotype control (n = 5, comparative analysis of the proportion of dead cells by Student’s *t*-test). **E**, **F** Tumor growth of subcutaneous SCCVII tumors **F** with or **E** without MEF treated by anti-PD-L1 antibody or isotype control (n = 5; mean ± SEM. Student’s* t*-test). **G** Representative pictures of immunohistochemical staining for CD8 and FoxP3. Scale bars = 50 µm. **H**, **I** The average number of CD8-positive or FoxP3-positive T cells in SCCVII tumors **H** with or **I** without MEF (n = 5, Student’s *t*-test). *P < 0.05, ^**^P < 0.01

SCCVII cells were derived from murine squamous cell carcinoma, and this allograft model can simulate esophageal squamous cell carcinoma. Immunofluorescence staining showed that anti-PD-L1 antibodies adhered to PD-L1-expressing CAFs, as DIG-PD-L1 stained αSMA^+^ cells (Fig. 6A and Supplemental Figure S10B). MFI of PD-L1 showed a notable decrease in both cancer cells and CAFs, indicating effective binding of the anti-PD-L1 antibody to cells expressing PD-L1 (Fig. 6B). The proportion of deceased cancer cells and CAFs exhibited a significant increase three days after administration of the anti-PD-L1 antibody, in comparison to the control groups (Fig. 6C, D). Next, the efficacy of the PD-L1 antibody was tested using the allograft model. In the group where SCCVII and MEF cells were co-inoculated, the administration of the anti-PD-L1 antibody resulted in a significant inhibition of tumor growth when compared to the group treated with isotype IgG (Fig. 6E, F). In evaluation of host tumor immunity, CD8^+^ T cells were also significantly increased, whereas Tregs were considerably decreased in the anti-PD-L1 group (Fig. 6G, H). In the SCCVII model, neither CD8^+^ T cells nor Tregs showed significant changes with the treatment (Figs. 6I and supplemental figure S11B). Similar to the MC38 + MEF models, these results indicate that anti-PD-L1 antibodies respond significantly to CAFs-rich tumors and enhance tumor immunity in SCCVII + MEF models.

## Discussion

We demonstrated that PD-L1 expression in CAFs and cancer cells was associated with poor outcomes in patients with esophageal cancer. Additionally, the PD-L1^+^ CAFs group had a higher number of CAFs in the tumor, indicating poor prognosis because we previously reported that the proportion of CAFs in the tumor was significantly correlated with the outcomes in clinical studies [11, 12]. Furthermore, interactions between cancer cells and CAFs mutually upregulate PD-L1 expression in vitro and in vivo, resulting in tumor aggressiveness, particularly in CAFs-rich models. Administration of anti-PD-L1 antibodies to CAFs-rich tumors suppresses tumor growth and activates tumor immunity, therefore, PD-L1-expressing CAFs are promising as a beneficial predictor of outcomes in patients with esophageal cancer.

In contrast, some studies have reported that patients with PD-L1^+^ CAFs had better survival in the non-small-cell lung or triple-negative breast cancer [27, 28]. Our results suggest that PD-L1 expression in CAFs was less elevated in the experimental model of esophageal adenocarcinoma, yet in esophageal squamous cell carcinoma, PD-L1 expression in fibroblasts was increased between cancer cells and fibroblasts in vitro. These results suggest that the impact of PD-L1-expressing CAFs on survival varied depending on the carcinoma and histological types. Interestingly, in vivo models, the PD-L1^+^ CAFs population in CAFs-rich tumors was significantly increased compared with CAFs-poor models in squamous cell carcinoma (SCCVII) and adenocarcinoma models (MC38). Furthermore, anti-PD-L1 antibody treatment was effective in both the CAF-rich models. Therefore, as the expected effect occurred in the experimental model in squamous cell carcinoma and adenocarcinoma cells, anti-PD-L1 antibody treatment can be a novel therapy for PD-L1-expressing CAFs.

It has been reported that interferon-γ, IL6, C-X-C motif chemokine ligand (CXCL) 2, CXCL5, and TGF-β upregulate PD-L1 expression [27, 29–33]. However, this study showed that TGF-β, which is one of the most well-known cytokines that stimulate fibroblasts to induce CAFs [34], did not increase PD-L1 expression in CAFs. In this study, the CMs of cancer cells or direct interaction with cancer cells led to increased PD-L1 expression in CAFs. This is probably because various factors released by cancer cells are involved in crosstalk with CAFs since various cytokines and chemokines were released from various cytokines and chemokines [9, 12, 31, 35]. Therefore, our results suggest that an interaction between cancer cells and CAFs is important for upregulating PD-L1 expression in cancer cells.

In tumors with abundant PD-L1-expressing CAFs, tumor progression was markedly inhibited by anti-PD-L1 antibodies compared with CAF-poor tumor models. Actually, damaged cells in cancer cells and CAFs in tumors treated with the anti-PD-L1 antibody were increased compared with the control groups. This is probably because PD-L1-expressing CAFs could be injured by antibody-dependent cellular cytotoxicity or component-dependent cytotoxicity by an anti-PD-L1 antibody. Another reason was likely that the anti-PD-L1 antibody was sufficiently distributed in the tumor in the CAFs-rich models with upregulated PD-L1 expression. Since anti-PD-L1 antibodies are mainly distributed in normal tissue [36], the inadequate effect of anti-PD-L1 antibody treatment in CAFs-poor models was due to insufficient accumulation in the tumor. Additionally, the anti-PD-L1 antibody as an ICI also caused an antitumor effect. Due to CAFs depletion by these effects, immunosuppression [12] and disturbance of drug delivery [6, 37] induced by CAFs can be improved. Therefore, these characteristics of the anti-PD-L1 antibody led to significant antitumor efficacy in CAFs-rich tumor models, owing to the advantage of simultaneously targeting cancer cells and CAFs.

The scoring systems for CPS or tumor proportion score (TPS) have proven valuable in predicting the efficacy of ICIs such as pembrolizumab or nivolumab. In our study, we conducted separate evaluations of PD-L1 positive cells in both cancer cells and CAFs. Although the findings of this study cannot be directly extrapolated to the CPS due to its distinct evaluation criteria, it is reasonable to speculate that PD-L1^+^ CAFs might be prevalent among the cells in CPS, given the abundant CAF population. Thus, further investigations are warranted to explore the prognostic significance of CPS and the potential impact of PD-L1^+^CAFs in the context of ICI therapy.

This study had some limitations. First, the evaluation of clinical specimens for patients with esophageal cancer was limited to a single institution. Therefore, a worldwide multicenter study is needed for universal analysis. Second, it was difficult to directly extrapolate in vivo data for esophageal cancer in syngeneic mice because mouse-derived esophageal cancer cells could not be obtained commercially. Third, we evaluated in vivo PD-L1 expression levels and the efficacy of the anti-PD-L1 antibody using only subcutaneous allograft tumor models. Orthotopic tumor models superiorly reflect the TME and immune landscape [38].

In conclusion, we demonstrated that PD-L1-expressing CAFs led to poor outcomes in clinical specimens in vitro and in vivo, resulting in tumor immunosuppression. Since the anti-PD-L1 antibody suppressed PD-L1-expressing CAFs and induced additional antitumor effects, the potential of PD-L1-expressing CAFs as biomarkers of ICIs should be validated. Therefore, PD-L1-expressing CAFs could be good targets for cancer therapy to inhibit tumor progression and improve host tumor immunity.

### Supplementary Information

Below is the link to the electronic supplementary material.

Supplementary file1 (DOCX 7515 kb)

## Data Availability

The datasets generated during and/or analysed during the current study are available from the corresponding author on reasonable request.
