# Supplementary material for: PD-L1-expressing cancer-associated fibroblasts induce tumor immunosuppression and contribute to poor clinical outcome in esophageal cancer
Source: Cancer Immunol Immunother. 2023 Sep 5;72(11):3787–802. doi: 10.1007/s00262-023-03531-2 (PMC10576702; doi:10.1007/s00262-023-03531-2)
Supplement: Supplementary file 1 — Supplementary file1 (DOCX 7515 kb) [file 262_2023_3531_MOESM1_ESM.docx]

Supplementary Information for

**PD-L1-expressing cancer-associated fibroblasts induce tumor immunosuppression and contribute to poor clinical outcome in esophageal cancer**

**Authors:** Kento Kawasaki^1^, Kazuhiro Noma^1^, Takuya Kato^1^, Toshiaki Ohara^1,2^, Shunsuke Tanabe^1^, Yasushige Takeda^1^, Hijiri Matsumoto^1^, Seitaro Nishimura^1^, Tomoyoshi Kunitomo^1^,

Masaaki Akai^1^, Teruki Kobayashi^1^, Noriyuki Nishiwaki^1^, Hajime Kashima^1^, Naoaki Maeda^1^, Satoru Kikuchi^1^, Hiroshi Tazawa^1,3^, Yasuhiro Shirakawa^1,4^, and Toshiyoshi Fujiwara^1^

**Affiliations:**

^1^ Department of Gastroenterological Surgery, Okayama University Graduate School of Medicine, Dentistry and Pharmaceutical Sciences, Okayama, Japan.

^2^ Department of Pathology & Experimental Medicine, Okayama University Graduate School of Medicine, Dentistry and Pharmaceutical Sciences, Okayama, Japan.

^3^ Center for Gene and Cell Therapy, Okayama University Hospital, Okayama, Japan.

^4^ Department of Surgery, Hiroshima City Hiroshima Citizens Hospital

*Corresponding author. Kazuhiro Noma, Department of Gastroenterological Surgery, Okayama University Graduate School of Medicine, Dentistry, and Pharmaceutical Sciences, 2-5-1 Shikata-cho, Kita-ku, Okayama 700-8558, Japan Phone: +81-86-235-7255; Fax: +81-86-221-8775; E-mail: [knoma@md.okayama-u.ac.jp](mailto:knoma@md.okayama-u.ac.jp)

**List of Supplementary Information**

Supplementary Figure S1. Representative pictures of PD-L1 expression in the stromal area

Supplementary Figure S2. Comparison of immune cells between PD-L1+/- cancer cell groups

Supplementary Figure S3. Survival curves for the variance of PD-L1 expression

Supplementary Figure S4. Gating strategy and evaluation of PD-L1 expression

Supplementary Figure S5. Dot plots of co-culture models using Cytotell UltraGreen

Supplementary Figure S6. PD-L1 expression in fibroblasts and OE33 cells stimulated by the cancer-conditioned medium of esophageal adenocarcinoma cell lines

Supplementary Figure S7. PD-L1 expression in FEF3 stimulated by TGF-β or conditioned medium of esophageal squamous cell carcinoma cells.

Supplementary Figure S8. Gating strategy and evaluation for PD-L1 in cancer cells and CAFs in vivo models

Supplementary Figure S9. Representative pictures of immunohistochemical staining for CD8, FoxP3, and αSMA in tumor tissues

Supplementary Figure S10. Digoxigenin-labeled anti-PD-L1 antibody administration for murine subcutaneous tumors

Supplementary Figure S11. Representative pictures of immunohistochemical staining for CD8 and FoxP3 in MC38 and SCCⅦ tumors without MEF

Supplementary Table S1. Clinicopathological features for PD-L1 in cancer cells

Supplementary Table S2. Univariate and multivariate analysis for overall survival

Supplementary Table S3. Univariate and multivariate analysis for relapse-free survival

Supplementary Table S4. Univariate and multivariate analysis for PD-L1 expression in cancer cells

**Supplemetary Figure S1**

**
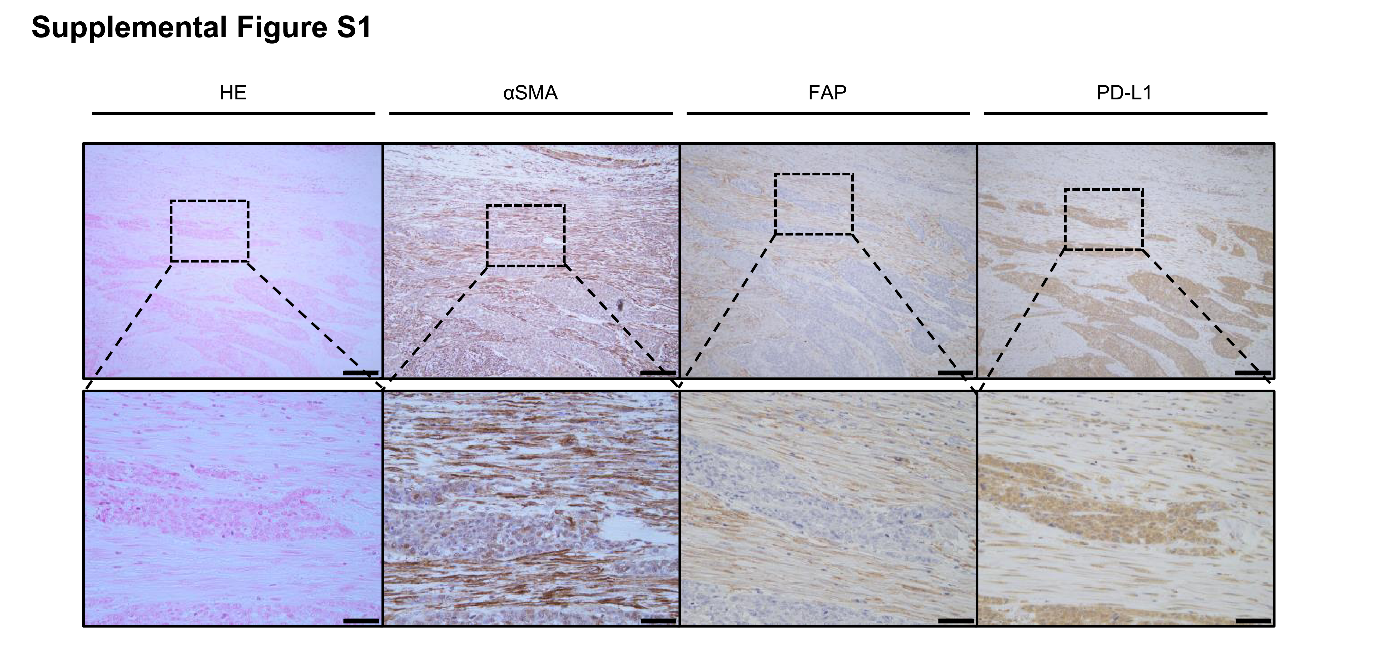
**

**Supplementary Figure S1.** **Representative pictures of PD-L1 expression in the stromal area**

Representative images of hematoxylin and eosin staining, α-SMA, FAP, and PD-L1 immunostaining. Scale bars = 100 µm. The lower figures are enlarged images. Scare bars = 50 µm.

**Supplemetary Figure S2**

**
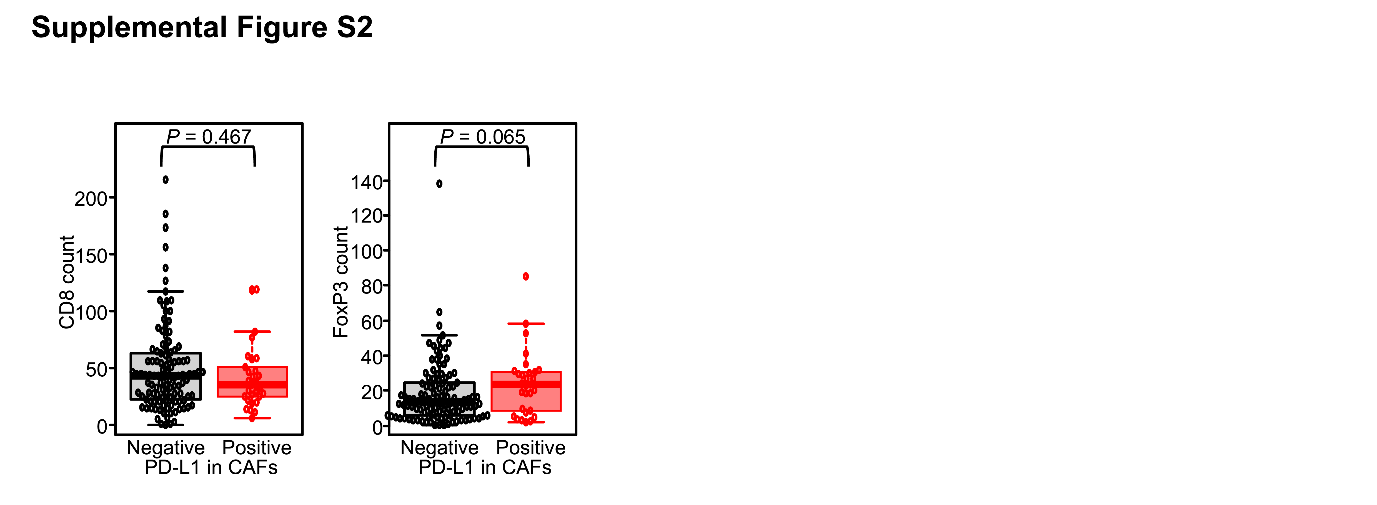
**

**Supplementary Figure S2. Comparison of immune cells between PD-L1^+/-^ cancer cell groups**

Comparison of CD8^+^ and FoxP3^+^ cells between PD-L1^+/-^ CAFs groups. Mann–Whitney U test.

**Supplemetary Figure S3**

**
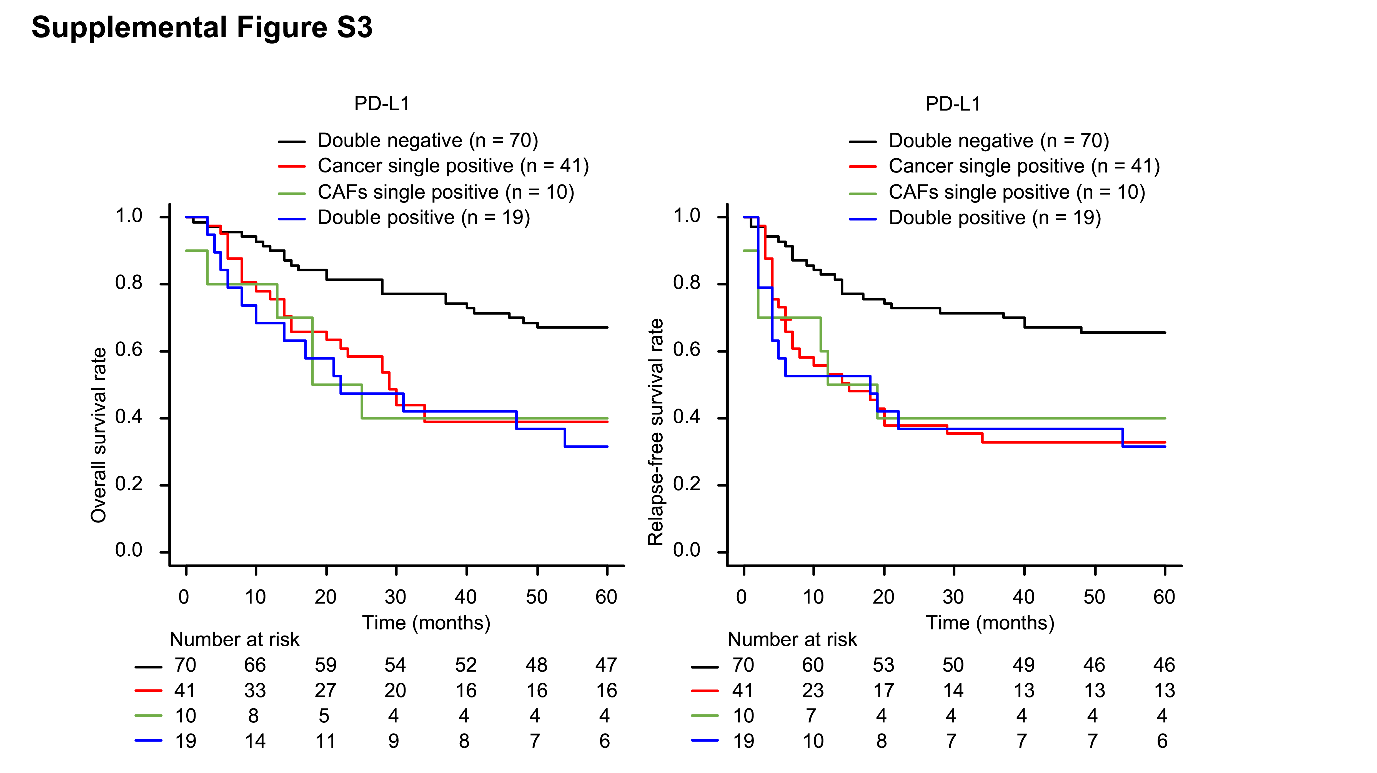
**

**Supplementary Figure S3.** **Survival curves for the variance of PD-L1 expression**

Survival curve according to the variance of PD-L1 expression analyzed using the Kaplan–Meier method (n = 140).

**Supplemetary Figure S4**

**
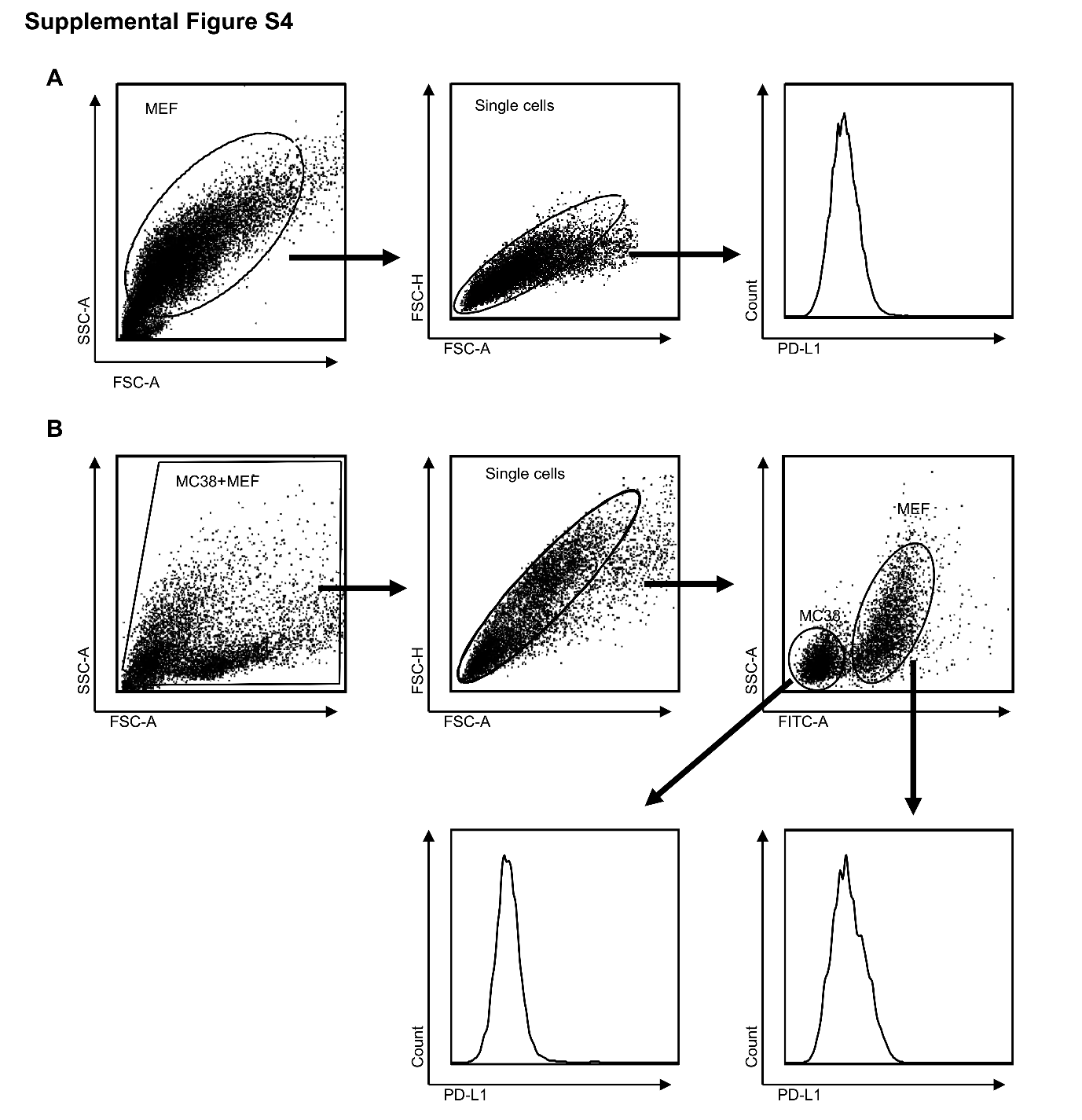
**

**Supplementary Figure S4. Gating strategy and evaluation of PD-L1 expression**

Gating strategy and representative histogram via flow cytometry of (A) fibroblasts activated by conditioned media and (B) cancer cells and fibroblasts activated in co-culture models.

**Supplemetary Figure S5**

**
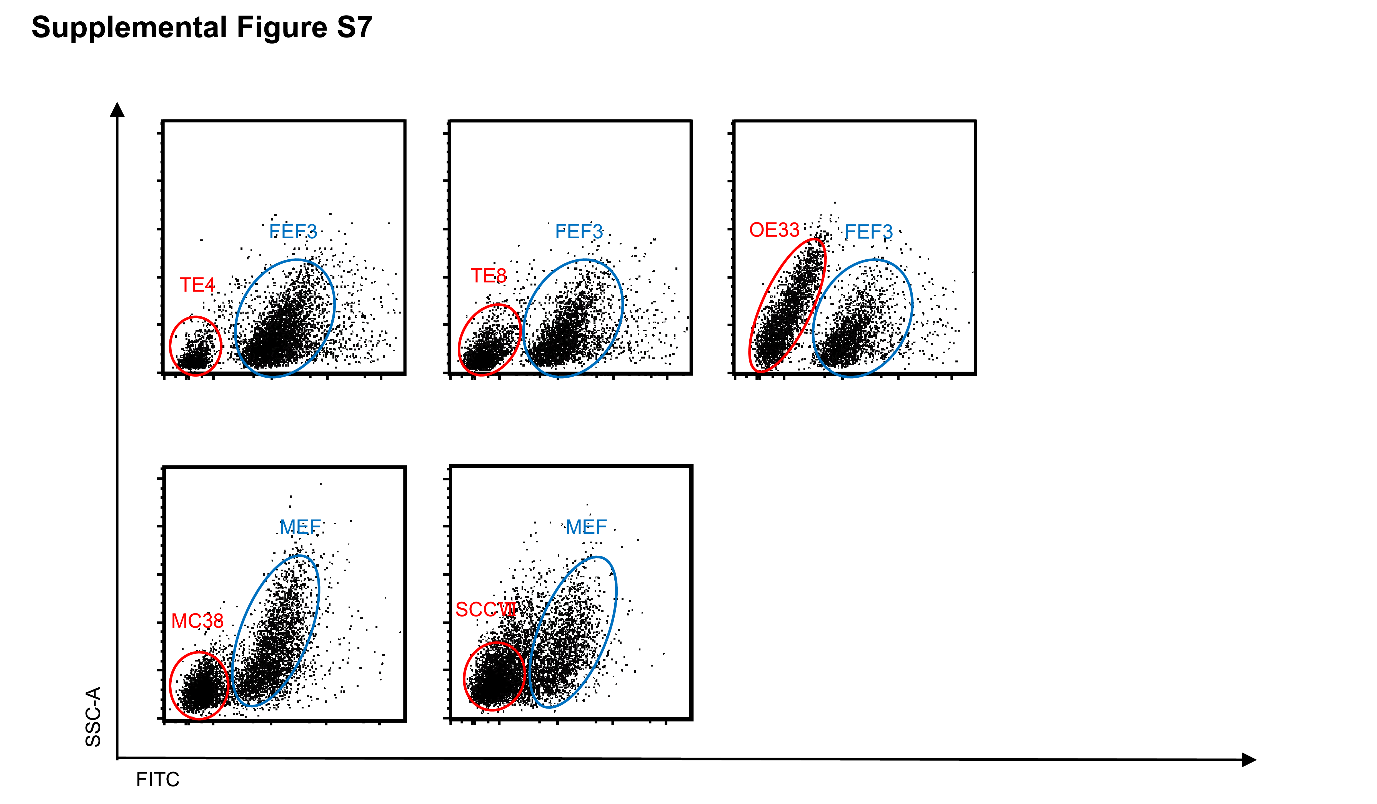
**

**Supplementary Figure 5. Dot plots of co-culture models using Cytotell UltraGreen**

Representative dot plots by flow cytometric analysis. Fibroblasts were detected using FITC and distinguished from cancer cells by pre-staining with ultra-green.

**Supplemetary Figure S6**

**
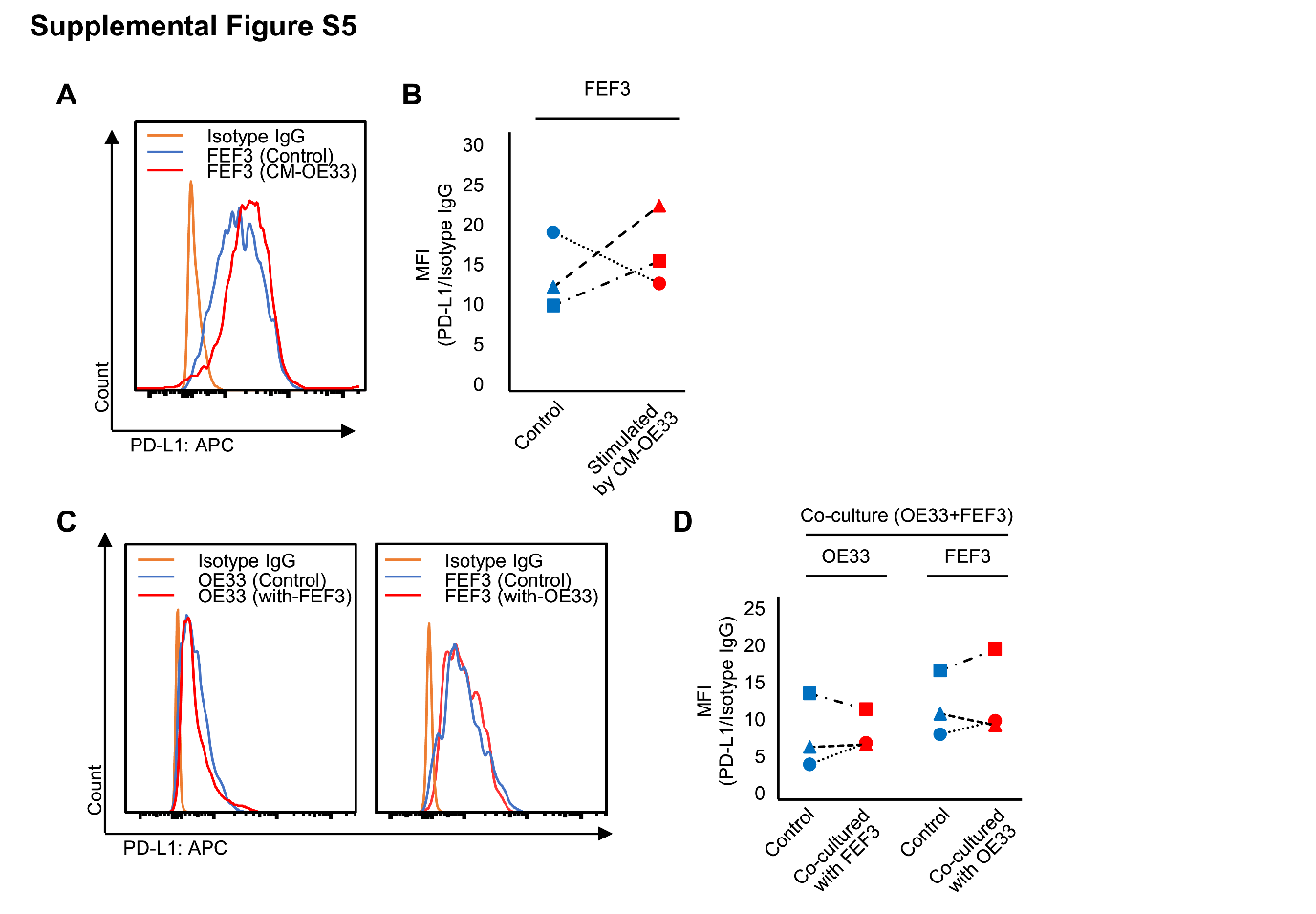
**

**Supplementary Figure S6.** **PD-L1 expression in fibroblasts and OE33 cells stimulated by the cancer-conditioned medium of esophageal adenocarcinoma cell lines**

(A, B) Flow cytometry analysis of cell surface PD-L1 expression in fibroblasts with or without activation by conditioned medium from OE33 cells. (A) Histogram and (B) comparison of PD-L1 expression. (C, D) Flow cytometry analysis of cell surface PD-L1 expression in OE33 cells and FEF3 in a co-culture model. (C) Histogram and (D) comparison of PD-L1 expression. n = 3, comparative analysis of mean fluorescence intensities using paired *t*-test.

**Supplemetary Figure S7**

**
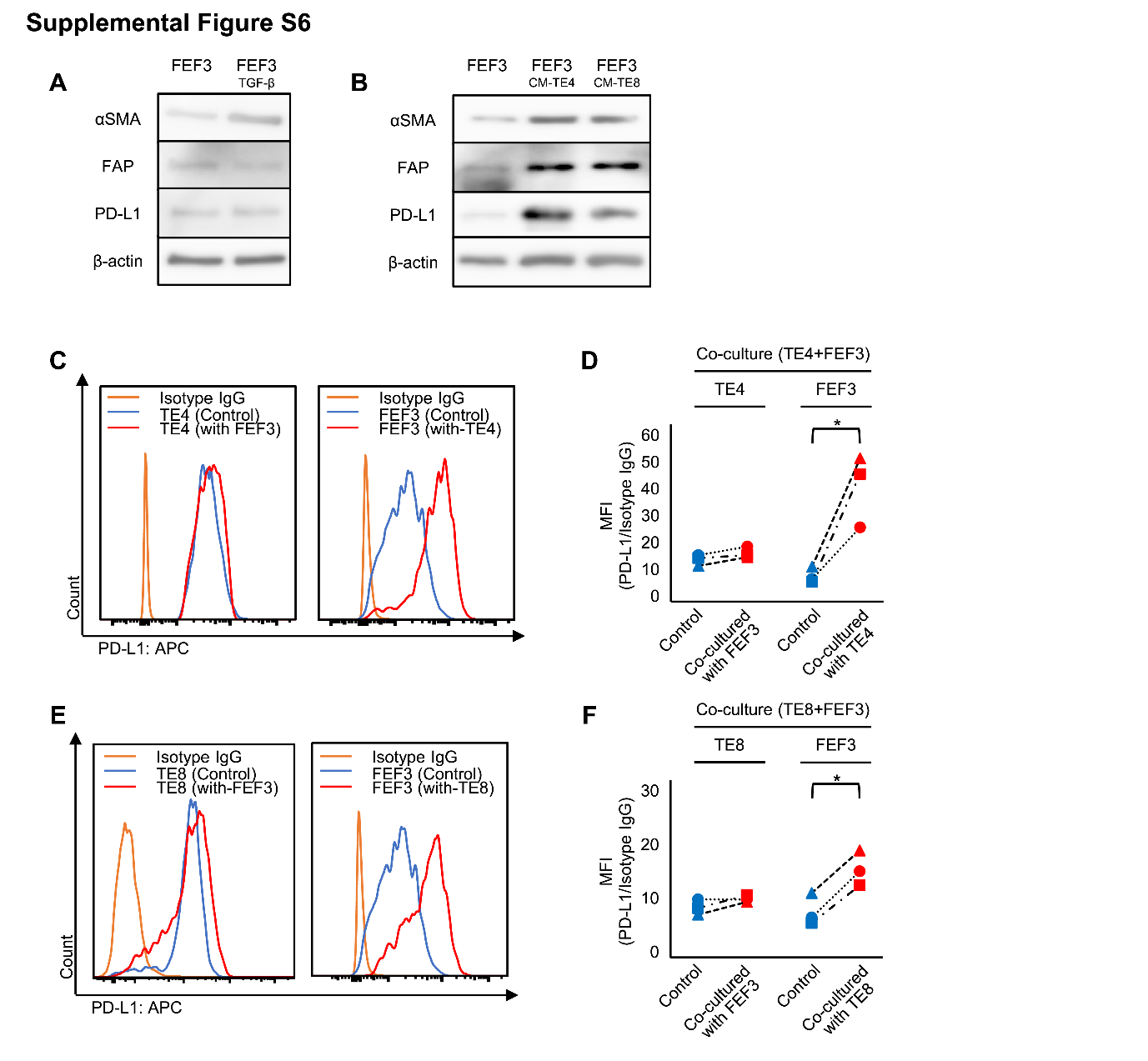
**

**Supplementary Figure S7.** **PD-L1 expression in FEF3 stimulated by TGF-β or conditioned medium of esophageal squamous cell carcinoma cells**

Western blotting was performed as described previously. The following antibodies were used; monoclonal anti-E-cadherin (#3195 clone, 24E10, Cell Signaling Technology), monoclonal anti-vimentin (#5741, clone D21H3, Cell Signaling Technology), monoclonal anti-αSMA (#19245, clone D4K9N, Cell Signaling Technology), polyclonal anti-FAP (ab53066, Abcam), monoclonal anti-PD-L1 (#13684, clone E1L3N, Cell Signaling Technology), and monoclonal anti-β-actin (A5441, clone AC-15, Sigma-Aldrich). The membranes were visualized using an Amersham Imager 600 (GE Healthcare, Little Chalfont/ UK). (A, B) FEF3 activated by (A) TGF-β and (B) conditioned medium of TE4 and TE8 subjected to western blotting of αSMA, FAP, PD-L1, and β-actin expression. (C, D) Flow cytometry analysis of cell surface PD-L1 expression in human cancer cells and fibroblasts in a co-culture model of TE4 and FEF3. (C) Histogram and (D) comparison of PD-L1 expression. (E, F) Flow cytometry analysis of cell surface PD-L1 expression in human cancer cells and fibroblasts in a co-culture model of TE8 and FEF3. (E) Histogram and (F) comparison of PD-L1 expression. n = 3, comparative analysis of mean fluorescence intensities by ratio paired t-test, *P < 0.05.

**Supplemetary Figure S8**

**
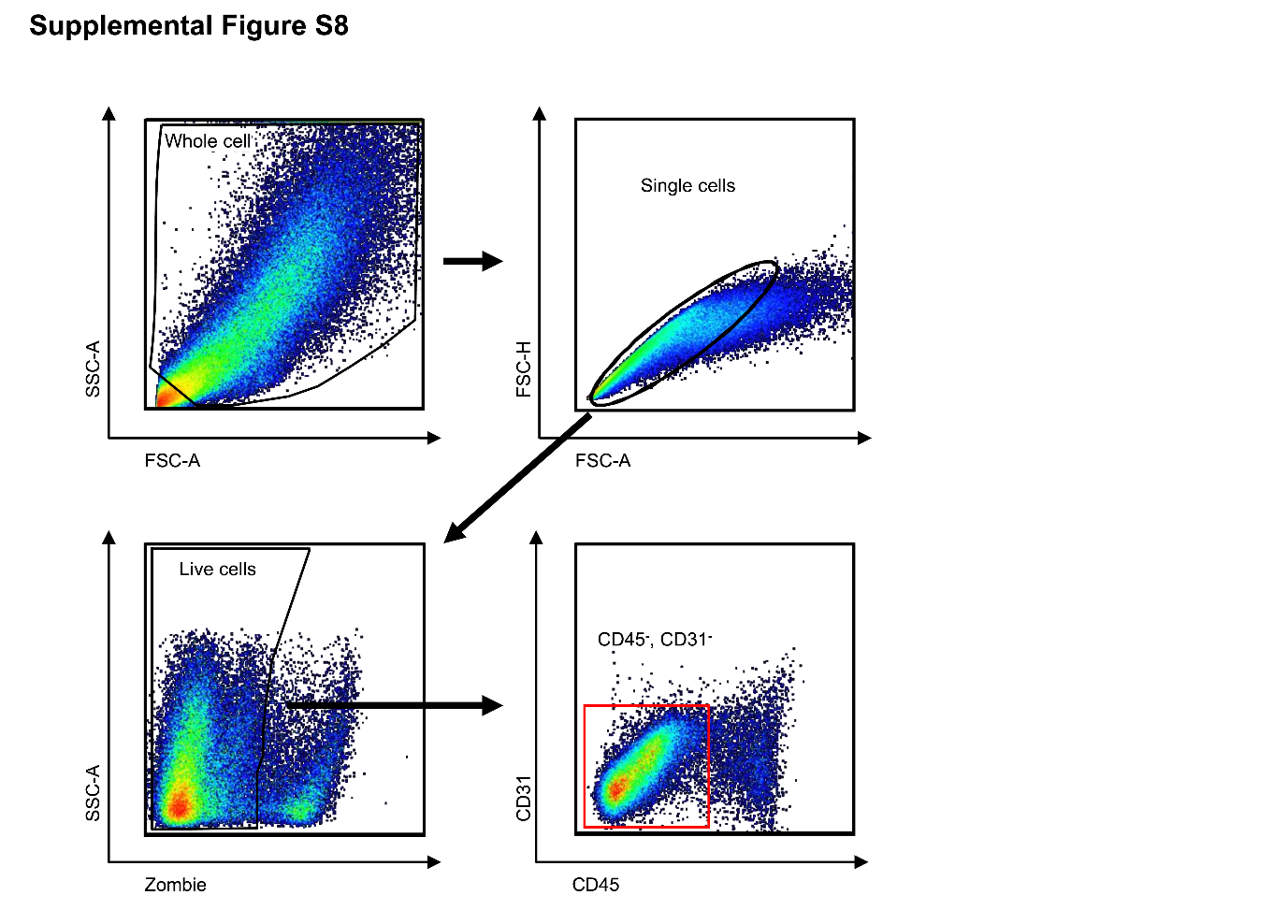
**

**Supplementary Figure S8. Gating strategy and evaluation for PD-L1 in cancer cells and CAFs in vivo models**

Gating strategy and representative flow cytometry plots. we carried out dead cell removal and subsequently gated out CD45 and CD31. The CD90.2 positive cells were identified as CAFs, while the CD90.2 negative cells were identified as cancer cells.

**Supplemetary Figure S9**


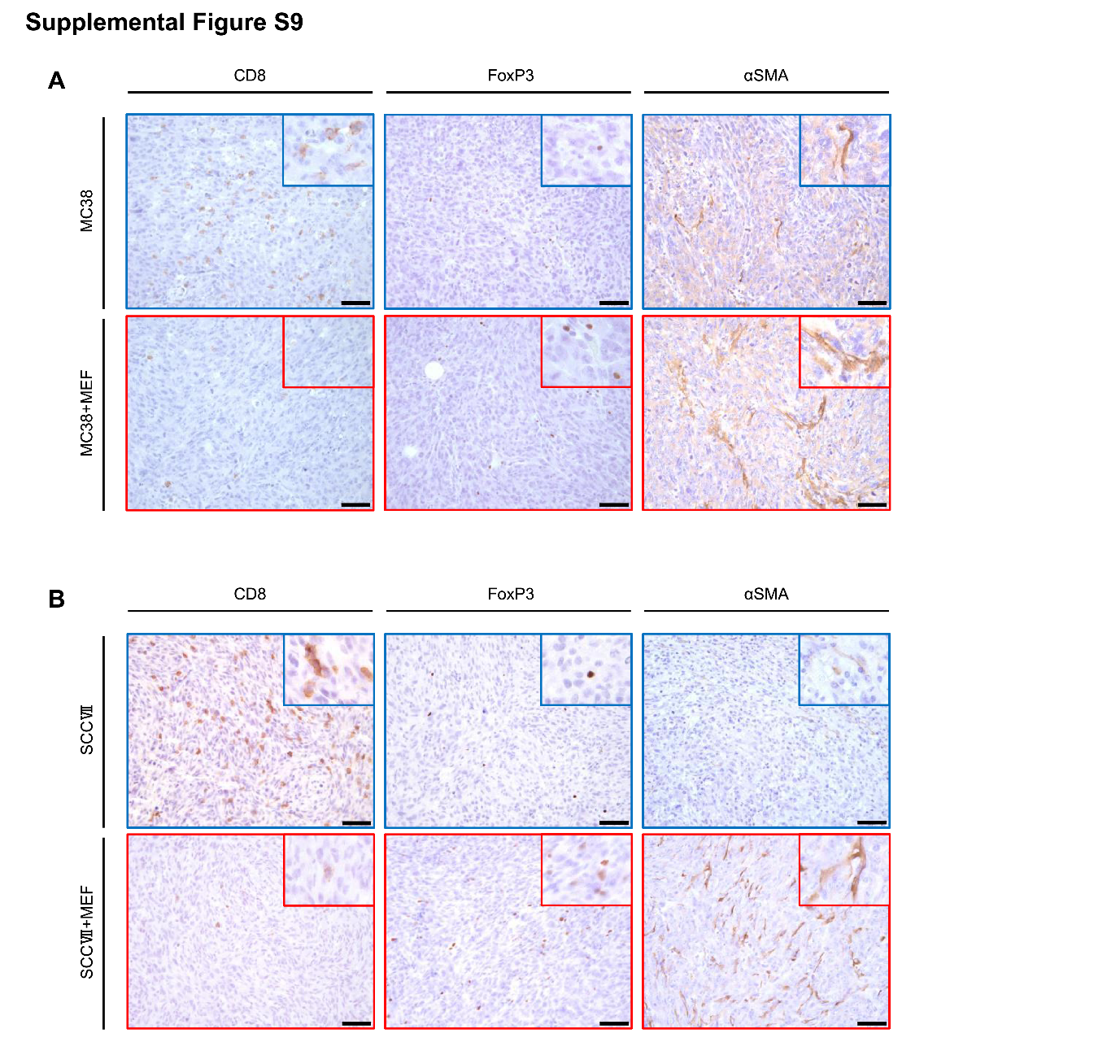


**Supplementary Figure S9.** **Representative pictures of immunohistochemical staining for CD8, FoxP3, and αSMA in tumor tissues**

(A) MC38 cells with and without MEF tumors. (B) SCCⅦ with or without MEF tumors. Scale bars = 50 µm.

**Supplemetary Figure S10**

**
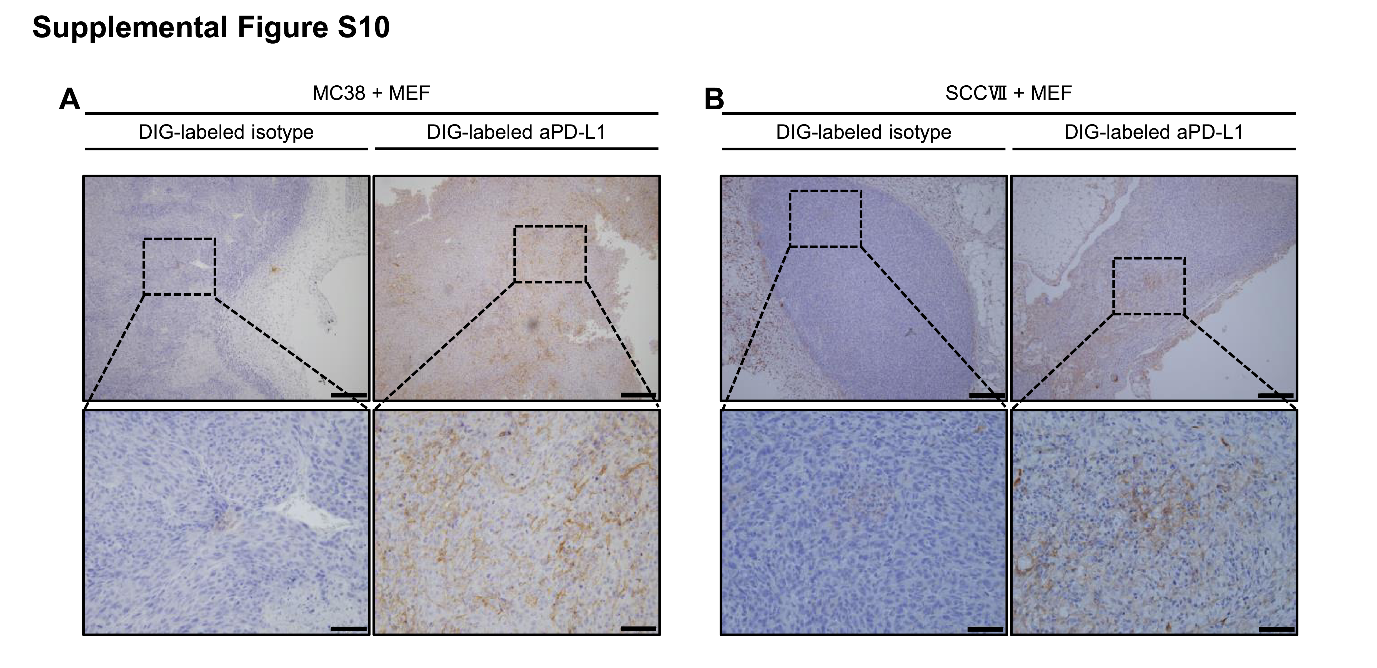
**

**Supplementary Figure S10.** **Digoxigenin-labeled anti-PD-L1 antibody administration for murine subcutaneous tumors**

(A, B) Representative pictures of immunohistochemical staining for Digoxigenin (DIG)-labeled anti-PD-L1 antibody (aPD-L1) and DIG-labeled isotype rat IgG2b. (A) MC38+MEF. (B) SCCⅦ+MEF. Scale bars = 200 µm. Lower figures are enlarged images. Scare bars = 50 µm.

**Supplemetary Figure S11**


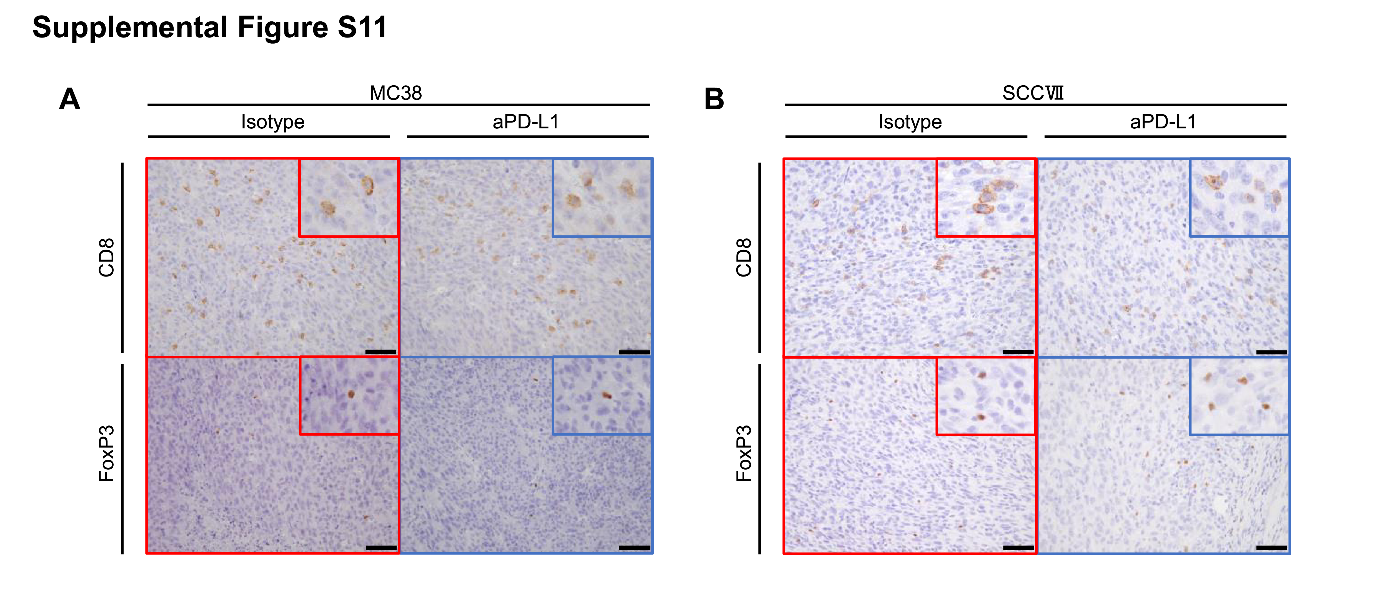
**Supplementary Figure S11.** **Representative pictures of immunohistochemical staining for CD8 and FoxP3 in MC38 and SCCⅦ tumors without MEF**

1. MC38 tumor without MEF. (B) SCCⅦ tumor without MEF. Scale bars = 50 µm.

**Supplementary Table S1.** **Clinicopathological features for PD-L1 in cancer cells**

|  | **Total** | **PD-L1(+)** | **PD-L1(-)** | ***P* value** |
| --- | --- | --- | --- | --- |
| **Variable** | **n = 140** | **n = 60 (42.9%)** | **n = 80 (57.1%)** |  |
| Age (years) | 67 (40–85) | 67 (44–85) | 67 (40–84) | 0.644§ |
| Sex (male/female) |  |  |  | 0.626† |
| Male | 121 (86.4%) | 53 (88.3%) | 68 (85.0%) |  |
| Female | 19 (13.6%) | 7 (11.7%) | 12 (15.0%) |  |
| Neo-adjuvant chemotherapy | 35 (25.0%) | 18 (30.0%) | 17 (21.2%) | 0.245† |
| Tumor location |  |  |  | 0.089† |
| Cervical | 12 (8.6%) | 4 (6.7%) | 8 (10.0%) |  |
| Upper | 24 (17.1%) | 5 (8.3%) | 19 (23.8%) |  |
| Middle | 60 (42.9%) | 30 (50.0%) | 30 (37.5%) |  |
| Lower | 28 (20.0%) | 15 (25.0%) | 13 (16.2%) |  |
| Abdominal | 16 (11.4%) | 6 (10.0%) | 10 (12.5%) |  |
| Pathological T stage |  |  |  | < 0.001* |
| T1 | 62 (44.3%) | 14 (23.3%) | 48 (60.0%) |  |
| T2 | 15 (10.7%) | 7 (11.7%) | 8 (10.0%) |  |
| T3 | 59 (42.1%) | 38 (63.3%) | 21 (26.2%) |  |
| T4 | 4 (2.9%) | 1 (1.7%) | 3 (3.8%) |  |
| Pathological N stage |  |  |  | 0.054† |
| N0 | 68 (48.6%) | 23 (38.3%) | 45 (56.2%) |  |
| N1 | 38 (27.1%) | 16 (26.7%) | 22 (27.5%) |  |
| N2 | 21 (15.0%) | 12 (20.0%) | 9 (11.2%) |  |
| N3 | 13 (9.3%) | 9 (15.0%) | 4 (5.0%) |  |
| Histological type |  |  |  | 0.422† |
| Squamous cell carcinoma | 123 (87.9%) | 55 (91.7%) | 68 (85.0%) |  |
| Adenocarcinoma | 12 (8.6%) | 3 (5.0%) | 9 (11.2%) |  |
| Other | 5 (3.6%) | 2 (3.3%) | 3 (3.8%) |  |
| αSMA Area Index | 7.76 (0.49–40.30) | 12.83 (1.39–33.25) | 4.73 (0.49–40.30) | < 0.001§* |
| FAP Area Index | 6.04 (0.01–39.91) | 8.54 (0.5–37.0) | 3.97 (0.01–39.91) | < 0.001§* |
| CD8 | 40.13 (0.25–215.75) | 43.38 (0.25–185.25) | 38.13 (0.50–215.75) | 0.931§ |
| FoxP3 | 15.13 (0.5–138) | 20.75 (2.0–138) | 11.86 (0.50–52.5) | 0.001§* |

Values are presented as median or n (%)

Mann–Whitney *U* test: §, Fisher’s exact test: †, **P* < 0.05 (statistical significance)

SMA, smooth muscle actin; FAP, fibroblast activation protein; FoxP3, forkhead box p3; PD-L1, programmed cell death ligand 1

**Supplementary Table S2.** **Univariate and multivariate analysis for overall survival**

|  |  | **Univariate analysis** | | |  | **Multivariate analysis** | | |
| --- | --- | --- | --- | --- | --- | --- | --- | --- |
| **Variable** | **Unfavorable/**  **favorable** | **HR** | **95% CI** | ***P* value** |  | **HR** | **95% CI** | ***P* value** |
| Age (years) | ≥70/＜70 | 1.09 | 0.67–1.79 | 0.720 |  |  |  |  |
| Sex | Male/Female | 2.91 | 1.06–8.00 | 0.039* |  | 2.88 | 1.04–7.97 | 0.041* |
| Neoadjuvant  chemotherapy | Yes/No | 2.20 | 1.33–3.63 | 0.002* |  | 1.50 | 0.88–2.54 | 0.132 |
| Pathological T stage | T2, T3, T4/T1 | 2.55 | 1.51–4.31 | < 0.001* |  | 1.46 | 0.79–2.69 | 0.226 |
| Pathological N stage | N1, N2, N3/N0 | 2.71 | 1.61–4.54 | < 0.001* |  | 2.01 | 1.15–3.55 | 0.015* |
| PD-L1 in cancer cells | Positive/Negative | 2.22 | 1.37–3.61 | 0.001* |  | 1.72 | 1.03–2.87 | 0.039* |

Cox proportional hazard model, **P* < 0.05 (statistical significance)

HR, hazard ratio; CI, confidence interval; PD-L1, programmed cell death 1.

Multivariate analysis was performed on statistically significant parameters obtained from the univariate.

**Supplementary Table S3.** **Univariate and multivariate analysis for relapse-free survival**

|  |  | **Univariate analysis** | | |  | **Multivariate analysis** | | |
| --- | --- | --- | --- | --- | --- | --- | --- | --- |
| **Variable** | **Unfavorable/favorable** | **HR** | **95% CI** | ***P* value** |  | **HR** | **95% CI** | ***P* value** |
| Age (years) | ≥70/＜70 | 0.97 | 0.60–1.57 | 0.892 |  |  |  |  |
| Sex | Male/Female | 3.06 | 1.11–8.40 | 0.030* |  | 2.79 | 1.01–7.74 | 0.048* |
| Neoadjuvant chemotherapy | Yes/No | 2.76 | 1.68–4.53 | < 0.001* |  | 1.58 | 0.88–2.81 | 0.122 |
| Pathological T stage | T2, T3, T4/T1 | 2.84 | 1.70–4.76 | < 0.001* |  | 2.05 | 1.21–3.49 | 0.008* |
| Pathological N stage | N1, N2, N3/N0 | 2.72 | 1.64–4.49 | < 0.001* |  | 2.14 | 1.27–3.60 | 0.004* |
| PD-L1 in cancer cells | Positive/Negative | 2.41 | 1.50–3.88 | < 0.001* |  | 2.02 | 1.22–3.34 | 0.006* |

Cox proportional hazard model, **P* < 0.05 (statistical significance)

HR, hazard ratio; CI, confidence interval; PD-L1, programmed cell death 1.

Multivariate analysis was performed on statistically significant parameters obtained from the univariate.

**Supplementary Table S4.** **Univariate and multivariate analysis for PD-L1 expression in cancer cells**

|  |  | **Univariate analysis** | | |  | **Multivariate analysis** | | |
| --- | --- | --- | --- | --- | --- | --- | --- | --- |
| **Variable** | **Unfavorable/favorable** | **OR** | **95% CI** | ***P*-value** |  | **OR** | **95% CI** | ***P*-value** |
| Age (years) | ≥70/＜70 | 1.04 | 0.52–2.07 | 0.920 |  |  |  |  |
| Sex | Male/Female | 1.34 | 0.49–3.63 | 0.570 |  |  |  |  |
| Neo-adjuvant chemotherapy | Yes/No | 1.59 | 0.74–3.43 | 0.239 |  |  |  |  |
| Pathological T stage | T2, T3, T4/T1 | 4.93 | 2.34–10.40 | < 0.001* |  | 2.29 | 0.87–6.02 | 0.093 |
| Pathological N stage | N1, N2, N3/N0 | 2.07 | 1.05–4.09 | 0.037* |  | 0.73 | 0.30–1.78 | 0.489 |
| Area index of αSMA | Positive/Negative | 1.83 | 1.08–3.10 | 0.024* |  | 4.72 | 1.81–12.30 | 0.001* |

Logistic regression analysis, **P* < 0.05 (statistical significance)

OR, odds ratio; CI, confidence interval; SMA, smooth muscle actin.

Multivariate analysis was performed on statistically significant parameters obtained from the univariate.
